# Process Optimization of *Solidago canadensis* Extracts: Impact on Polyphenolic Profile, Antioxidant Capacity, and Cytotoxic Activity

**DOI:** 10.3390/antiox15060737

**Published:** 2026-06-10

**Authors:** Cornelia Fursenco, Violeta Alexandra Ion, Oana-Crina Bujor, Simona Marcu Spinu, Mihaela Dragoi Cudalbeanu, Ionela Avram, Liliana Bădulescu, Alina Orțan, Tatiana Calalb, Livia Uncu

**Affiliations:** 1 Department of Pharmacognosy and Pharmaceutical Botany, Scientific Centre for Drug Research, Faculty of Pharmacy, Nicolae Testemițanu State University of Medicine and Pharmacy, Malina Mica Street 66, MD-2025 Chisinau, Moldova; cornelia.fursenco@usmf.md (C.F.); tatiana.calalb@usmf.md (T.C.); 2 Research Center for Studies of Food Quality and Agricultural Products, University of Agronomic Sciences and Veterinary Medicine of Bucharest, 59 Marasti Blvd, 011464 Bucharest, Romania; oana.bujor@qlab.usamv.ro (O.-C.B.); liliana.badulescu@qlab.usamv.ro (L.B.); 3 Faculty of Land Reclamation and Environmental Engineering, University of Agronomic Sciences and Veterinary Medicine of Bucharest, 59 Marasti Blvd, 011464 Bucharest, Romania; simona.spinu@fifim.ro (S.M.S.); mihaela.dragoi@fifim.ro (M.D.C.); alina.ortan@fifim.ro (A.O.); 4 Department of Genetics, Faculty of Biology, University of Bucharest, 1–3 Aleea Portocalelor, 060101 Bucharest, Romania; ionela.avram@unibuc.ro; 5 Department of Pharmaceutical and Toxicological Chemistry, Scientific Centre for Drug Research, Faculty of Pharmacy, Nicolae Testemițanu State University of Medicine and Pharmacy, Malina Mica Street 66, MD-2025 Chisinau, Moldova; livia.uncu@usmf.md

**Keywords:** flavonoids, microwave-assisted extraction, thermal maceration, antioxidant activity, cytotoxicity

## Abstract

Optimizing the extraction of bioactive compounds from *Solidago* sp. is essential for the development of plant-derived products with therapeutic and nutraceutical potential. Microwave-assisted (MW) and thermal maceration (TM) extraction of *S. canadensis* aerial parts were comparatively investigated to maximize total flavonoid content (TFC). The obtained extracts were subsequently freeze-dried for storage prior to chemical and biological analyses. Extraction conditions were optimized using a Box–Behnken design. Chemical characterization was performed by FTIR, HPLC-PDA, LC-MS/MS, and GC-MS, enabling detailed profiling of phenolic compounds and terpenoids. Antioxidant capacity was assessed using the DPPH radical scavenging assay, while cytotoxic activity was evaluated against HepG2, HCT-8, and HT-29 tumor cell lines, with HEK-293 cells used as a non-tumorigenic control cell line. Multivariate analysis (PCA) was applied to establish relationships between phytochemical composition and biological responses. Higher TFC values were obtained using MW extraction, whereas TM extracts exhibited greater antioxidant activity. Both extract types induced selective cytotoxic effects against tumor cell lines, while maintaining negligible toxicity toward normal HEK-293 cells. PCA revealed distinct clustering patterns between MW and TM extracts and confirmed a strong association between phenolic composition and bioactivity. The combination of optimized extraction, freeze-drying, and integrated chemical–biological evaluation produced *S. canadensis* extracts with well-defined phytochemical profiles and biological activity, supporting their potential use in nutraceutical, and pharmaceutical applications.

## 1. Introduction

*Solidago canadensis* L. (family Asteraceae), commonly known as Canadian goldenrod, is a perennial herbaceous species native to North America that was introduced to Europe in the early seventeenth century, primarily for ornamental purposes. Following its introduction, *S. canadensis* became naturalized in various European habitats, colonizing disturbed areas such as abandoned agricultural land and riverbanks, and rapidly becoming invasive [1,2,3]. In Romania, *S. canadensis* has acquired invasive status due to the presence of humid habitats and favorable climatic conditions that promote its rapid spread [4]. In contrast, in the Republic of Moldova, this species is considered synanthropic and is mainly cultivated for ornamental use; moreover, due to the predominantly dry steppe conditions, it does not currently pose a significant risk of invasiveness [5].

The therapeutic value of *S. canadensis* has been recognized for centuries. In European phytotherapy, the herbal material of this species has traditionally been used in the management of urinary and genital disorders. Furthermore, its widespread use in modern medicine is attributed to its complex profile of specialized metabolites, particularly polyphenolic compounds, which are responsible for its documented antioxidant, antimicrobial, and anti-inflammatory activities [2,3,5]. It is noteworthy that this plant is included in the European Pharmacopoeia (Ph. Eur. 12.3) under the monograph *Solidaginis herba*, which requires a minimum flavonoid content of 2.5%, expressed as hyperoside [6].

From a phytochemical perspective, *S. canadensis* is recognized as a valuable source of phenolic compounds, including flavonoids, phenolic acids, and tannins [1,2,3]. These compounds play an important role in the plant’s ecological success by enhancing defense mechanisms against pathogens and contributing to biodiversity conservation [1,2,3,7]. In addition to their ecological functions, these metabolites are associated with a wide range of pharmacological effects and have demonstrated efficacy in the treatment of inflammatory urinary tract disorders, arthritis and rheumatic conditions, wound healing, and mild hypertension [1,2,8,9,10]. At the same time, there is growing interest in the isolation of these compounds from plant sources as safe, natural, and cost-effective alternatives to synthetic antioxidants, some of which have been associated with toxic effects [11].

In recent years, a growing number of studies have focused on the chemical composition and quantitative profile of phenolic compounds in *S. canadensis*, employing advanced physicochemical techniques. For example, in the Istria region of Croatia, LC–MS analysis of leaf and flower extracts revealed that hydroxycinnamic acids and flavonols are the predominant classes of phenolic compounds, with 5-caffeoylquinic acid identified as the major constituent in leaves and quercetin-3-rutinoside predominating in flowers [2]. Similarly, a study conducted in Slovakia using LC–MS/MS showed that ethanol extracts contained the highest levels of phenolic compounds, particularly rutin, quercetin, quercitrin, and chlorogenic acid. These findings collectively underscore the considerable pharmacological potential of *S. canadensis* and highlight the need for further research on the bioavailability, efficacy, and safety of its bioactive constituents, suggesting that this invasive species may represent a valuable resource for pharmaceutical development [3].

For chemical analysis, extraction represents a critical initial step, ensuring the isolation and purification of bioactive compounds from plant materials. Accordingly, recent research has focused on the development and optimization of various methods for the extraction of polyphenolic compounds [12]. It has been demonstrated that several factors, including the extraction technique, solvent selection, solubility of bioactive compounds, extraction temperature and duration, and solvent-to-sample ratio, play a crucial role in determining both the yield and quality of phenolics obtained from plant extracts [13,14].

Given the structural diversity of polyphenolic compounds, establishing a universal, standardized extraction method capable of efficiently recovering most polyphenols from different plant sources remains challenging [15]. Conventional extraction techniques, such as maceration, percolation, and Soxhlet extraction, are associated with several limitations, including low extraction yields, prolonged processing times, high solvent consumption, and negative environmental impacts [14,16]. In recent years, novel techniques such as microwave-assisted extraction (MW), supercritical fluid extraction (SFE), and ultrasound-assisted extraction (UAE) have been increasingly investigated [14,16,17].

Among these, MW is a relatively recent technique that employs microwave energy to rapidly heat the solvent and plant matrix, resulting in enhanced mass transfer and disruption of plant cell walls, thereby facilitating the release of intracellular compounds such as polyphenols, flavonoids, alkaloids, and other secondary metabolites. According to the literature, this innovative approach offers several advantages, including reduced solvent consumption, shorter extraction times, and improved extract quality. Moreover, it aligns with the principles of green chemistry by promoting the use of environmentally friendly solvents and minimizing the ecological impact associated with large-scale solvent consumption [17,18,19,20]. In terms of performance, recent studies consistently report that MW achieves higher yields of phenolic and other bioactive compounds in significantly shorter extraction times compared to conventional methods. This improvement is mainly attributed to rapid volumetric heating and intensified mass transfer induced by microwaves [21,22]. In contrast, conventional extraction techniques, although slower, may still be advantageous when working with highly thermolabile compounds because it allows milder and more controlled heating conditions, reducing the risk of degradation [23]. Although microwave-assisted extraction (MW) and traditional maceration (TM) have been individually investigated in numerous studies, systematic comparisons under optimized extraction conditions remain limited. In this context, the present study provides a direct comparison between MW and TM under optimized parameters, aiming to highlight differences in extraction efficiency and bioactive compound recovery.

Another important stage in pharmaceutical research is the optimization of extraction methods. The optimization of parameters such as solvent type, solvent-to-sample ratio, temperature, and extraction time is essential to ensure maximal recovery of bioactive phenolic compounds [24]. Determining optimal extraction conditions enables the production of standardized, phenolic-rich extracts suitable for pharmaceutical, nutraceutical, and functional food applications, thereby contributing to the valorization of this species as a sustainable source of bioactive compounds [25].

In addition to extraction optimization, appropriate post-extraction preservation techniques are equally important for maintaining the stability, integrity, and biological activity of phytochemical compounds, particularly in phenolic-rich plant extracts. Recent studies have shown that freeze-drying is more effective than conventional drying methods in preserving total phenolic content, flavonoid stability, and antioxidant activity, thereby ensuring higher retention of phytochemical integrity and biological functionality in plant matrices [26,27,28]. Due to these advantages, lyophilization is considered one of the most suitable pre-treatment methods for studies focusing on the extraction and evaluation of bioactive compounds from plant materials [26,27].

Furthermore, determining the content and potential availability of bioactive constituents in *S. canadensis* extracts is an essential requirement, given their relevance in phytomedicine and prospective applications in the food industry. These considerations further emphasize the therapeutic potential of *S. canadensis* and the necessity for continued research addressing the bioavailability, pharmacological efficacy, and safety of its bioactive compounds, supporting the valorization of this invasive species for pharmaceutical development.

Despite extensive research on the chemical composition and biological activities of *S. canadensis*, limited attention has been given to the optimization of extraction methods aimed at maximizing the recovery of polyphenolic compounds from its aerial parts. Although the phytochemical profile of *S. canadensis* has been increasingly investigated, the relationship between extraction strategy, preservation approach, and the resulting biological activity remains insufficiently explored. The present study addresses this gap by developing a comprehensive and integrated approach combining extraction optimization, freeze-drying preservation, and bioactivity evaluation to obtain lyophilized extracts with enhanced flavonoid content, suitable for antioxidant and cytotoxic assessments.

Extraction conditions were optimized using both thermal maceration and microwave-assisted techniques, followed by concentration and freeze-drying to produce powders. In addition, a comparative evaluation of the two extraction techniques was performed, alongside a systematic investigation of the relationship between chemical composition and biological activity (antioxidant and cytotoxic effects). This combined strategy provides new insights into composition-driven bioactivity and supports the development of standardized *S. canadensis* extracts with potential applications in pharmaceutical, nutraceutical, and functional food formulations.

## 2. Materials and Methods

### 2.1. Plant Material and Sample Preparation

**Figure 1:**
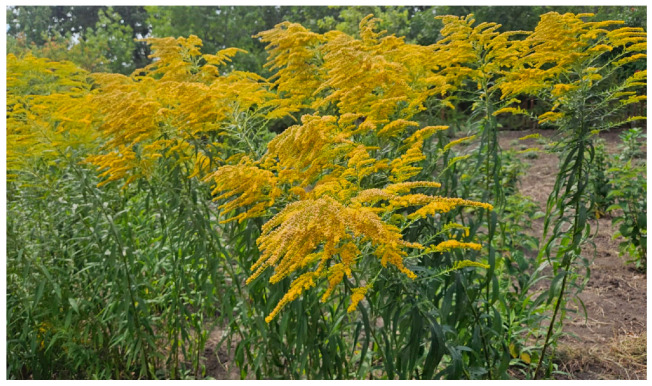
*S. canadensis* plant from the collection of the Scientific-Practical Center in the Field of Medicinal Plants, Republic of Moldova (original photo).

The aerial parts (herba) of *S. canadensis* (Canadian goldenrod), consisting of stems, leaves, and flowers, were used as plant material in the present study. Samples were collected during the full flowering stage, when approximately 70% of the flowers were open, between late July and early August 2024. The plant material used for analysis consisted of a mixture of samples collected during three independent harvesting events. The harvesting was carried out at the collection of the Scientific-Practical Center in the Field of Medicinal Plants (46°54′06″ N, 28°40′05″ E), Nicolae Testemitanu State University of Medicine and Pharmacy (Figure 1). Botanical identification was performed according to established taxonomic criteria. Voucher specimens of *Solidago canadensis* L. (SC 237249) were authenticated and deposited in the Herbarium of the *Alexandru Ciubotaru* National Botanical Garden (Institute) of the Moldova State University, Republic of Moldova.

Following collection and authentication, the plant material was transferred to the phytochemical laboratory repository of the Department of Pharmacognosy and Pharmaceutical Botany, Faculty of Pharmacy, Nicolae Testemițanu State University of Medicine and Pharmacy. For storage, the samples were kept under controlled conditions, with regulated temperature, humidity, and protection from light to preserve phytochemical integrity. The material was maintained on shelving units in breathable, labeled kraft paper bags, ensuring adequate ventilation and preventing moisture accumulation.

For the extraction procedures, the dried aerial parts were ground using a Grindomix GM 200 laboratory mill (Retsch GmbH, Haan, Germany). The grinding process was carried out for a total duration of 60 s, consisting of three cycles of 20 s each, at a rotational speed of 8000 rpm. The resulting material was subsequently sieved to obtain a homogeneous powder with a particle size of 0.5 mm and stored in paper bags under controlled environmental conditions until further analysis.

### 2.2. Optimization of Total Flavonoid Content Using Thermal Maceration and Microwave-Assisted Extraction

#### 2.2.1. Box–Behnken Design (BBD) for Extraction Optimization

The optimization of the flavonoid extraction process was performed using the Response Surface Methodology (RSM) with the Design-Expert^®^ software package, version 13.0.5.0. The study was designed based on a Box–Behnken design model.

The experimental design was randomized, without blocks, and included a total of 17 experimental runs. The mathematical model applied to describe the relationship between independent factors and response was a quadratic one. Within this experimental design, three independent continuous factors were investigated: *temperature (°C)*, *plant to solvent ratio* (w/v), and *solvent concentration (ethanol to water ratio, %)*. Factor levels were coded in the range [−1, +1], corresponding to the minimum and maximum limits of each experimental parameter. The independent variables and their levels are presented in Table 1, while the seventeen experimental runs are summarized in Table 2. The response variable studied was *total flavonoid content (TFC)*.

Thermal maceration was carried out using a UN 110 laboratory oven (Memmert GmbH + Co. KG, Schwabach, Germany). Accurately weighed quantities of powdered plant material were mixed with the appropriate volume of hydroethanolic solvent according to the experimental design (Table 2). The extraction was performed at the selected temperatures for 2 h under controlled conditions. This method facilitated the diffusion of bioactive compounds from the plant matrix into the solvent through thermal enhancement of mass transfer processes.

Microwave-assisted extraction was performed using an Ethos Easy microwave extraction system (Milestone^™^ SpA, Sorisole, Italy). The plant material was mixed with the extraction solvent according to the predefined plant material-to-solvent ratios and subjected to microwave irradiation at a power of 600 W for 1 h. Microwave-assisted extraction enhances extraction efficiency through rapid heating, improved solvent penetration, and disruption of plant cell structures, facilitating the release of intracellular bioactive compounds. Following extraction, the obtained extracts were filtered under vacuum using a vacuum filtration system to remove insoluble plant residues.

#### 2.2.2. Total Flavonoid Content

The total flavonoid content (TFC) of the hydroethanolic extracts was determined using the aluminum chloride colorimetric method [29,30]. Analyses were performed on the extracts prior to solvent evaporation in order to assess extraction efficiency under different experimental conditions and to identify the optimal parameters for flavonoid recovery. Briefly, 1.2 mL of diluted extract was mixed with 1.2 mL of a 2% (w/v) AlCl_3_ solution. After mixing, the solution was incubated at room temperature for 60 min in the dark to allow complex formation. Absorbance was measured against a blank at 425 nm using a UV–Vis spectrophotometer (Specord 210 Plus, Analytik Jena GmbH, Jena, Germany). A calibration curve (y = 0.0176x + 0.0071, R^2^ = 0.998) was prepared using quercetin as a standard, and results were expressed as mg quercetin equivalents (QE) per g dry weight (mg QE/g DW). All measurements were performed in triplicate, and results are presented as mean ± standard deviation.

### 2.3. Lyophilization and Phytochemical Characterization of S. canadensis Extracts

#### 2.3.1. Freeze-Drying Extracts

Following optimization, two separate *S. canadensis* extracts were obtained under the optimal conditions for microwave-assisted extraction (MW_opt_) and thermal maceration (TM_opt_). The filtrates were then concentrated under reduced pressure using a Laborota 4000 rotary evaporator (Heidolph Instruments GmbH & Co. KG, Schwabach, Germany) to remove the solvent and obtain concentrated extracts, and subsequently lyophilized to produce freeze-dried powders suitable for phytochemical characterization, antioxidant activity, and cytotoxicity analyses.

Freeze-drying was performed using a Christ LyoCube 4–8 lyophilizer (Martin Christ Gefriertrocknungsanlagen GmbH, Osterode am Harz, Germany). The extracts were frozen at −80 °C for 24 h and lyophilized under controlled conditions for 72 h at a condenser temperature of −55 °C and a vacuum pressure of 0.500 mbar. This process ensured the complete removal of residual solvent while preserving the structural integrity and stability of thermolabile bioactive compounds, particularly phenolic compounds and flavonoids. The obtained lyophilized extracts were collected, weighed to determine the extraction yield, and stored in airtight containers until analysis.

#### 2.3.2. FTIR Analysis of Phytochemical Compounds

Fourier Transform Infrared (FTIR) Spectroscopy was performed to identify the phytochemical functional groups in the *S. canadensis* powder extracts, using a JASCO FT-IR 6300 instrument (Jasco International Co., Ltd., Tokyo, Japan) equipped with a Specac ATR Golden Gate (Specac Ltd., Orpington, UK) with a KRS5 lens. *S. canadensis* powders were dissolved in methanol at a concentration of 10 mg/mL, filtered through 0.45 μm polytetrafluoroethylene (PTFE) microfilters (Corning, New York, NY, USA), and analyzed directly to obtain characteristic absorption spectra in the wavenumber range of 4000–400 cm^−1^. The spectral acquisition resolution was 4 cm^−1^, and each spectrum represented the average of 32 scans. Background spectra were recorded against air using the same parameters.

#### 2.3.3. GC-MS Analysis of Volatile and Semi-Volatile Compounds

The same *S. canadensis* powders, dissolved in methanol at a concentration of 10 mg/mL, were analyzed by Gas Chromatography coupled with Mass Spectrometry (GC-MS) to identify volatile and semi-volatile compounds. Chromatographic separation and mass spectrometry detection were performed using an Agilent 7820A GC system coupled to an Agilent 5977MS (Agilent Technologies, Santa Clara, CA, USA) equipped with an HP-5MS column (30 m × 250 μm × 0.25 μm, Agilent Technologies, Santa Clara, CA, USA). The oven temperature was initially set to 50 °C for 3 min, then ramped to 250 °C at 5 °C/min and held for 15 min. Helium was used as the carrier gas at a flow rate of 1 mL/min, and the GC injection (1 μL) was performed in split mode (1:20) at 280 °C. The electron impact (EI) ion source operated at 70 eV. The scan rate was 0.2 s/scan over a mass range of 40–500 amu. The ion trap temperature was 220 °C, while the MS injection and interface temperatures were set to 250 °C.

Identification of volatile and semi-volatile compounds was carried out using National Institute of Standards and Technology (NIST) reference spectra in combination with data from the literature.

#### 2.3.4. HPLC-PDA and LC-MS/MS Analysis of Phenolic Compounds

HPLC-PDA and LC-MS/MS were used as complementary analytical approaches serving different purposes. HPLC-PDA was employed for the targeted quantification of the major phenolic constituents for which authentic standards and UV-visible response were available, whereas LC-MS/MS was used for the broader tentative profiling of phenolic compounds based on accurate mass and fragmentation data, allowing the detection of minor, co-eluting, or structurally related constituents not adequately resolved by PDA alone.

Identification of the minor phenolic composition in the freeze-dried powders was carried out using Liquid Chromatography coupled with Mass Spectrometry (LC-MS/MS). Methanolic extracts (80% methanol) at a concentration of 10 mg/mL were analyzed to identify polyphenolic compounds. Chromatographic separation was performed on an Acquity UPLC CSH C18 column (1.7 μm, 2.1 mm × 150 mm), protected by an Acquity UPLC CSH C18 VanGuard pre-column (1.7 μm, 2.1 mm × 5 mm; Waters, Milford, MA, USA). The column temperature was maintained at 40 °C, while the autosampler was set to 15 °C, with an injection volume of 5 μL using an Acquity UPLC I-Class system (Waters, Milford, MA, USA).

Phenolic compounds were eluted using a binary solvent system consisting of water with 0.1% formic acid (A) and acetonitrile with 0.1% formic acid (B), applying a gradient from 5% to 100% B over 40 min at a flow rate of 0.2 mL/min. Detection was performed using an Xevo G3 QTof mass spectrometer (Waters, Milford, MA, USA) operating in negative-ion mode (ESI^−^), with collision energies of 10 and 30 eV. The scan range was set between 50 and 1600 *m*/*z*, with an acquisition rate of 5 spectra per second. Additional operating parameters included a drying gas temperature of 275 °C (10 L/min), sheath gas temperature of 325 °C (12 L/min), nebulizer pressure of 35 psi, capillary voltage of −4000 V, skimmer voltage of 65 V, and fragmentor voltage of 140 V. Minor phenolic compounds were tentatively identified based on accurate mass measurements, fragmentation patterns, and comparison with literature data.

For quantitative analysis, major phenolic compounds were quantified using an Agilent Technologies 1200 HPLC system (Agilent Technologies, Santa Clara, CA, USA) equipped with a UV–DAD detector. Separation was achieved on a Zorbax Eclipse Plus C18 column (150 mm × 4.6 mm i.d., 5 μm particle size; Agilent Technologies, Santa Clara, CA, USA). Identification of compounds was performed by comparing retention times and UV–Vis spectra with those of authentic standards. Quantification was carried out using calibration curves constructed from standard solutions in the linear range of 1–50 µg/mL.

Detection wavelengths were selected according to the absorption maxima of individual compounds: 325 nm for chlorogenic acid and neochlorogenic acid, and 350 nm for rutin hydrate, quercetin-3-O-glucopyranoside, quercetin-3-galactoside, kaempferol-3-O-rutinoside, and kaempferol-3-O-glucoside. Compounds lacking authentic standards were quantified as equivalents: those exhibiting maximum absorbance at 325 nm were expressed as chlorogenic acid equivalents, while those with maxima at 350 nm were expressed as rutin equivalents. Results were expressed as milligrams per gram of dry weight (mg/g DW).

#### 2.3.5. Antioxidant Activity

The free radical scavenging activity of the freeze-dried extracts was evaluated using 2,2-diphenyl-1-picrylhydrazyl (DPPH), following the method described by Marcu Spinu et al. (2024) [31]. Optimized extracts (MW_opt_ and TM_opt_) were diluted to a concentration of 4 mg/mL and mixed in a 1:1 ratio with DPPH solution (100 µg/mL). The mixtures were incubated in the dark for 30 min, after which the absorbance was measured at 517 nm using a FLUOstar^®^ Omega microplate reader (BMG LABTECH, Ortenberg, Germany). Results were expressed as milligram Trolox equivalents per gram of plant material (mg TE/g). IC50 values were obtained from dose–response curves using GraphPad Prism Software version 10.5.0.

#### 2.3.6. Cytotoxic Effects

The cytotoxicity of the optimized freeze-dried extracts was evaluated using the MTT (3-(4,5-dimethyl-2-thiazolyl)-2,5-diphenyl-2H-tetrazolium bromide) reduction assay on HEK-293 (embryonic kidney), HepG2 (liver tumor), HCT-8 (colorectal tumor), and HT-29 (colon tumor) cell lines (CLS Cell Lines Service GmbH, Eppelheim, Germany). HEK-293 cells were cultured in Fibroblast Medium (FM) (Innoprot, Derio, Spain), while HepG2 and HT-29 cells were grown in Dulbecco’s Modified Eagle Medium (DMEM) (Sigma-Aldrich, St. Louis, MO, USA). All culture media were supplemented with 10% Fetal Bovine Serum (FBS) (Sigma-Aldrich) and 1% penicillin-streptomycin (Sigma-Aldrich).

Cells were treated with extracts and incubated at 37 °C in a 5% CO_2_ atmosphere for 24 h. Stock solutions of the extracts (40 mg/mL in dimethyl sulfoxide, DMSO) were diluted 1:4 with Phosphate-Buffered Saline (PBS) to 10 mg/mL, and further diluted to final concentrations of 1000, 100, and 50 μg/mL in the culture medium. Hydrogen peroxide (H_2_O_2_) was used as a positive control, and 2.5% DMSO in PBS served as a negative control.

After incubation, the supernatants were removed, and cells were treated with 100 µg/mL MTT solution, followed by incubation at 37 °C for 4 h. The resulting purple formazan crystals were dissolved in 100 μL DMSO, and absorbance was measured at 570 nm using a Synergy HTX Multi-Mode Microplate Reader (Biotek, Winooski, VT, USA). Cytotoxicity was expressed as a percentage inhibition relative to the controls.

### 2.4. Statistical Analysis

All experiments were performed in triplicate (*n* = 3), and results are presented as mean ± standard deviation (SD). Statistical significance of the cytotoxicity data for the *S. canadensis* extracts was assessed using two-way ANOVA in GraphPad Prism software, version 10.5.0 (Boston, MA, USA). Post hoc comparisons were performed using Tukey’s multiple comparisons test to identify significant differences between means, with *p*-values < 0.05 considered statistically significant. The assumptions for parametric analysis (normality and homogeneity of variance) were checked prior to performing ANOVA.

## 3. Results

### 3.1. Optimization of Phenolic Compounds Extractions

The ethanolic extracts obtained by MW and TM exhibited total flavonoid content (TFC) ranging from 10.09 to 19.51 mg QE/g DW. The highest TFC was observed for MW (run 16), achieved at a temperature of 125 °C, a plant-to-solvent ratio of 1:30 (w/v), and a solvent concentration of 70%. The lowest TFC was also obtained by MW (run 10). The results for all experimental runs are summarized in Table 3.

#### 3.1.1. Statistical Analysis of Developed Optimization Model

The optimization of total flavonoid content as the response variable was modeled using Response Surface Methodology, considering temperature, plant-to-solvent ratio, and solvent concentration as independent factors. The Box–Behnken Design was employed because it efficiently evaluates nonlinear relationships between independent variables and the response while requiring fewer experiments than a full factorial design.

The results of the statistical analysis, obtained via Analysis of Variance (ANOVA), are presented in Table 4. The model summary includes the coefficient of determination (R^2^) and *p*-values for each extraction method studied. A quadratic model was applied to describe the relationship between independent factors and TFC, allowing the assessment of linear effects, interactions between factors, and quadratic effects.

The resulting models revealed very low probability values (*p* < 0.001), indicating a highly significant model. Terms with *p*-values less than 0.05 were considered statistically significant. For microwave-assisted extraction (MW), the significant model terms were A (temperature), B (plant-to-solvent ratio), AB, AC, A^2^, and B^2^, whereas C (solvent concentration), BC, and C^2^ were not significant. This suggests that solvent concentration did not have a notable effect on TFC in MW.

For thermal maceration, the significant terms were A, B, C, AB, AC, A^2^, and C^2^, while BC and B^2^ were not significant.

For both extraction methods, the *p*-value for lack of fit was greater than 0.05, indicating that the lack of fit was not significant. The predicted R^2^ values were in reasonable agreement with the adjusted R^2^ values, with differences less than 0.2, confirming the reliability of the models. Adequate Precision, which measures the signal-to-noise ratio, indicated an adequate signal in both cases. Notably, the quadratic effect of temperature was significant for both extraction methods.

The model equations, expressed in terms of coded factors (A, B, and C), were used to predict TFC for specific levels of each independent factor and to evaluate the relative impact of the factors by comparing their coefficients. The model equations for TFC in *S. canadensis* extracts are as follows: for TM (Equation (1)) and for MW (Equation (2)).

(1) TPC_TM_ (mg QE/g) = 15.37 + 0.2363A + 1.79B + 0.4837C + 1.28AB + 0.6425AC + 0.08BC − 0.3179A^2^ − 0.1355B^2^ − 0.4930C^2^

(2) TPC_MW_ (mg QE/g) = 18.59 + 0.8845A + 0.7588B − 0.1153C − 2.16AB + 1.72AC − 0.31BC − 1.75A^2^ − 2.67B^2^ + 0.35C^2^

#### 3.1.2. 3D Response Surface Modeling

**Figure 2:**
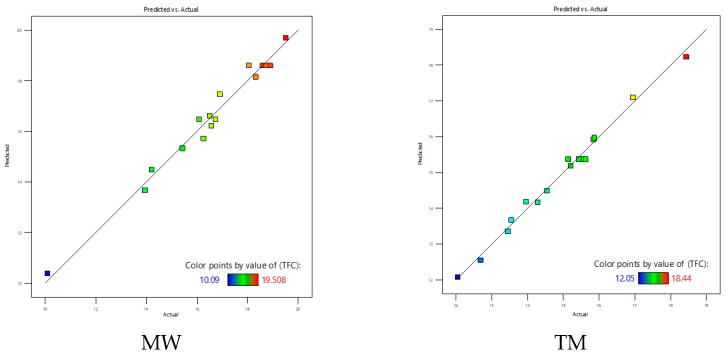
Correlation plot of predicted vs. experimental TFC values.

Analysis of the response surface plots was performed to evaluate the influence of the independent factors—temperature, plant-to-solvent ratio, and solvent concentration—on the dependent factor, total flavonoid content. The suitability of the generated model for TFC optimization was confirmed by the strong correlation between the experimental values and those predicted by the model (Figure 2).

**Figure 3:**
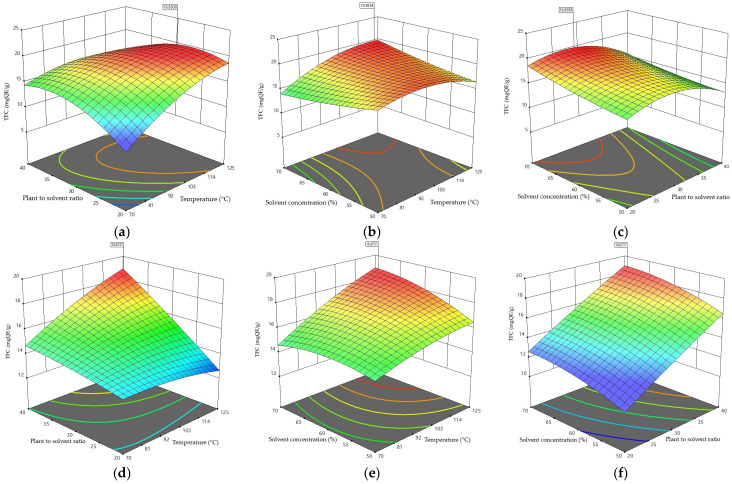
3D response surface plots illustrating the effects of the studied factors on TFC. The color scale from red to blue represents the maximum and minimum TFC values, respectively. (**a**) MW—temperature vs. plant to solvent ratio. (**b**) MW—temperature vs. solvent concentration. (**c**) MW—plant to solvent ratio vs. solvent concentration. (**d**) TM—temperature vs. plant to solvent ratio. (**e**) TM—temperature vs. solvent concentration. (**f**) TM—plant to solvent ratio vs. solvent concentration.

This correlation indicates that the model satisfactorily describes the relationship between the analyzed variables—both independent and dependent—demonstrating its ability to predict system behavior within the investigated experimental domain. Additionally, the RSM enabled efficient evaluation of both the individual effects of the process factors and their interactions on TFC. To provide a more detailed understanding of these interactions, the modeling results are presented as three-dimensional (3D) response surface plots, which illustrate the simultaneous influence of temperature, plant-to-solvent ratio, and solvent concentration on the studied response (TFC). The 3D plots are shown in Figure 3.

By analyzing the results for the maximization of TFC for the two extraction methods—MW and TM—the optimal conditions were identified for each. For MW, the optimal parameters were a temperature of 123 °C, a plant-to-solvent ratio of 1:29 (w/v), and a solvent concentration of 70%, resulting in a TFC of 19.89 mg QE/g DM and a desirability function of 1.0. For TM, the optimal conditions were a temperature of 125 °C, a plant-to-solvent ratio of 1:40 (w/v), and a solvent concentration of 69%, yielding a TFC of 18.87 mg QE/g DM and a desirability function of 1.0.

Comparison of these results shows that MW produced a slightly higher TFC than TM. The optimal parameters for both methods are relatively similar, with the main difference being the higher plant-to-solvent ratio required in TM. These findings suggest that MW is more efficient for flavonoid extraction, likely due to the combined effects of rapid heating and enhanced mass transfer facilitated by microwave radiation.

#### 3.1.3. Model Validation

Applying the optimized parameters, the extractions were repeated to validate the predictive capability of the developed model. The experimental values obtained under the optimal extraction conditions were then compared with the values predicted by the mathematical model, confirming its validity. The results are summarized in Table 5.

The results demonstrated a good agreement between the experimental values obtained under the optimal extraction conditions and those predicted by the model, confirming its capacity to accurately describe the relationship between the process variables and the analyzed response. The small differences observed between the experimentally determined and model-predicted values indicate that the developed model is reliable and can be confidently used to predict the total flavonoid content in *S. canadensis* extracts.

### 3.2. Phytochemical Profiles of Freeze-Dried Extracts

The extraction yield for the TM_opt_ method was 11.303 g of freeze-dried extract obtained from 31.5 g of plant material, corresponding to a yield of 35.88% (*w*/*w*). In comparison, the MW_opt_ method yielded 10.560 g of freeze-dried extract from the same amount of plant material, corresponding to a yield of 33.52% (*w*/*w*).

#### 3.2.1. FTIR Profile

**Figure 4:**
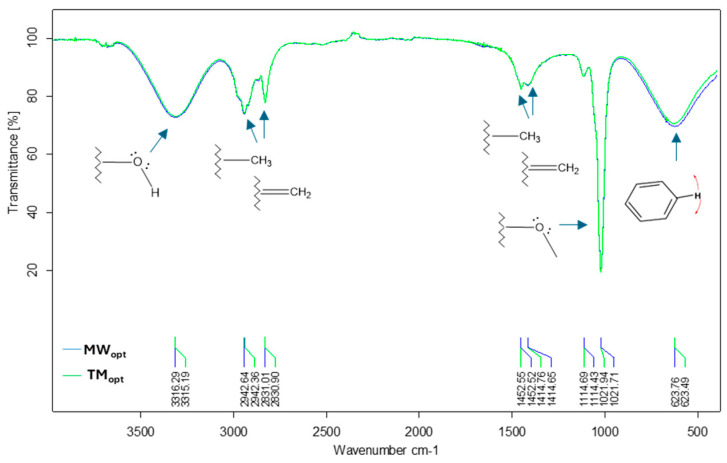
FTIR spectra of *S. canadensis* extracts.

The FTIR spectrum of *S. canadensis* extracts was used to identify the functional groups of phytochemicals based on peak assignments in the infrared region. The results for the most dominant FTIR peaks and functional groups are presented in Figure 4 and Table 6.

The extracts showed characteristic absorption bands at 3316 cm^−1^ (MW_opt_) and 3315 cm^−1^ (TM_opt_), indicative of O–H stretching vibrations of hydroxyl groups. Additional bands at 2831 cm^−1^ and 2942 cm^−1^ were attributed to C–H stretching vibrations of alkyl groups, including both asymmetric and symmetric stretching of –CH_2_ and –CH_3_ groups, confirming the presence of alkyl chains in the phytochemicals of both extracts. Bands at 1452 cm^−1^ and 1414 cm^−1^ correspond to C–H bending vibrations in aliphatic groups, further supporting the presence of alkyl compounds. The prominent bands at 1114 cm^−1^ and 1020 cm^−1^ are associated with C–O and C–O–C stretching vibrations, typical of alcohols and ethers. Lastly, signals at 623 cm^−1^ were attributed to out-of-plane C–H bending vibrations in phenolic rings, confirming the aromatic nature of *S. canadensis* phytochemicals.

#### 3.2.2. GC-MS Volatile and Semi-Volatile Compounds Profile

GC-MS analysis revealed 30 volatile and semi-volatile compounds in MW_opt_ extract, and 28 volatile and semi-volatile compounds in TM_opt_ extract (Table 7). Most compounds belonged to monoterpenes and oxygenated monoterpenes, such as D-limonene, (–)-cis-verbenol, verbenone (L), cis-carveol, carvone, bornyl acetate, endo-1,5,6,7-tetramethylbicyclo[3.2.0]hept-6-en-3-ol, 2-methyl-4-(2,6,6-trimethylcyclohex-1-enyl)but-2-en-1-ol, 2-butenal, and 2-methyl-4-(2,6,6-trimethyl-1-cyclohexen-1-yl)-. The analysis also identified sesquiterpenes and oxygenated sesquiterpenes, including spathulenol, caryophyllene oxide, 5β,7β-H,10α-eudesm-11-en-1α-ol, 6-epi-shyobunol, and platambin. Diterpenes and triterpenes such as andrographolide, phytol, and lupeol were present. Aromatic compounds and their derivatives, including catechol, 4-ethylcatechol, eugenol, 4-hydroxy-2-methylacetophenone, coumaran, 4-(2,4,4-trimethyl-cyclohexa-1,5-dienyl)-but-3-en-2-one, 3-hydroxymethylene-1,7,7-trimethylbicyclo[2.2.1]heptan-2-one, and 2-[4-methyl-6-(2,6,6-trimethylcyclohex-1-enyl)hexa-1,3,5-trienyl]cyclohex-1-en-1-carboxaldehyde were also identified. Fatty acids and their esters, including hexadecanoic acid methyl ester, linoleic acid methyl ester, linolenic acid methyl ester, and methyl stearate, were detected. Related to the methodology effect of the extraction parameters, the compound endo-1,5,6,7-tetramethylbicyclo[3.2.0]hept-6-en-3-ol is absent from the MW_opt_ extract, and D-limonene and 5β,7β-H,10α-eudesm-11-en-1α-ol are absent from the TM_opt_ extract.

#### 3.2.3. Phenolic Compounds Profile

The polyphenolic profile of the optimized *S. canadensis* extracts was characterized using complementary HPLC–PDA and LC-MS/MS analyses. HPLC–PDA was employed primarily for the quantification of major phenolic compounds, providing a reliable chemical fingerprint for quality assessment of the extracts obtained under optimized conditions (thermal maceration, TM_opt_, and microwave-assisted extraction, MW_opt_). In contrast, LC-MS/MS analysis enabled a more comprehensive profiling through the tentative identification of minor polyphenolic constituents based on their [M–H]^−^ ions and MS/MS fragmentation patterns, supported by comparison with literature data (Table 8).

A total of 11 polyphenolic compounds were characterized, including hydroxycinnamic acids (p-coumaric acid, ferulic acid, p-coumaroylquinic acid, and chlorogenic acid), a hydroxybenzoic acid (gallic acid), quinic acid, and flavonoids such as the flavanone naringenin, the flavone luteolin, and flavonols including quercetin, isorhamnetin, and rutin.

**Figure 5:**
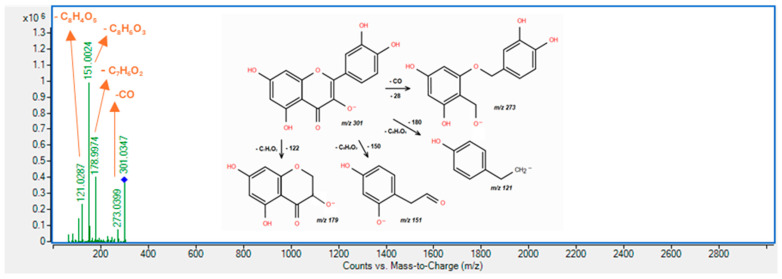
Fragmentation pathway of quercetin [M−H]^−^ at *m*/*z* 301.

Figure 5 illustrates the proposed MS/MS fragmentation pathway of quercetin by its deprotonated molecular ion [M−H]^−^ at *m*/*z* 301. Quercetin’s fragmentation is dominated by cleavage of the central C ring via a retro-Diels–Alder mechanism. Quercetin has a flavonol structure with two aromatic rings, A and B, joined by the heterocyclic ring C. In MS/MS, collision energy induces selective cleavage of the C ring, and the resulting fragments retain information about the molecule’s phenolic substituents. The *m*/*z* 179 ion is a major RDA fragment, and the *m*/*z* 151 ion is a highly diagnostic fragment for quercetin and its derivatives. In many quercetin glycosides, the carbohydrate moiety is lost first, resulting in the *m*/*z* 301 aglycone, which then fragments to *m*/*z* 179 and *m*/*z* 151. An additional fragment ion at *m*/*z* 273 may be associated with neutral losses of CO, whereas the fragment ion at *m*/*z* 121 may be related to further fragmentation of the aromatic moieties of the quercetin skeleton [45,46,47].

The quantitative analysis of polyphenolic compounds in the optimized *S. canadensis* extracts revealed that MW_opt_ and TM_opt_ were effective in recovering major hydroxycinnamic acids and flavonoids. In the MW_opt_ extracts, dicaffeoylquinic acid was the predominant compound, quantified at 8.09 ± 0.89 mg/g DW, followed by chlorogenic and neochlorogenic acids, totaling 5.94 ± 1.59 mg/g. Rutin hydrate was also abundant at 10.04 ± 0.71 mg/g DW, while other flavonoids, including quercetin-3-O-glucopyranoside, kaempferol-3-O-rutinoside, and isorhamnetin-3-O-rutinoside, were present in the range of 1.40–3.94 mg/g DW. The total polyphenol content of the MW_opt_ extracts was 49.96 ± 3.66 mg/g DW, with relative standard deviations generally below 15%, indicating good reproducibility for the major compounds.

In the TM_opt_ extracts, dicaffeoylquinic acid was slightly higher at 8.51 ± 0.42 mg/g DW, while chlorogenic and neochlorogenic acids were quantified at 7.13 ± 0.63 mg/g DW. Rutin hydrate reached 10.78 ± 0.95 mg/g DW, and the other flavonoids ranged from 1.30 to 6.41 mg/g DW. The total polyphenol content of TM_opt_ extracts was 54.48 ± 4.08 mg/g DW, slightly higher than in the MW_opt_. While the TM_opt_ method enabled enhanced extraction of minor polyphenolic compounds, the variability of some minor components was higher, with relative standard deviations reaching up to 21%.

### 3.3. Antioxidant Activity of Optimized Freeze-Dried Extracts

**Figure 6:**
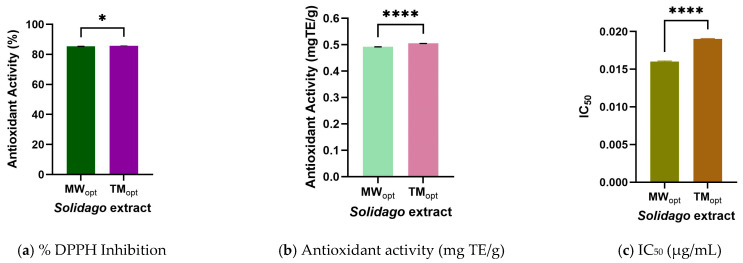
Antioxidant activity of optimized *S. canadensis* extracts. Significant differences were noted at the following levels: * = *p* < 0.05 and **** = *p* < 0.0001.

The antioxidant activity of the two optimized extracts, obtained using different extraction methods (MW and TM), was evaluated using the stable free radical DPPH. The results are presented in Figure 6.

According to the results obtained, the percentage of DPPH inhibition was relatively constant, indicating a high and stable level of antioxidant activity (MW_opt_ = 85.36 ± 0.18%, TM_opt_ = 85.73 ± 0.09%, *p* = 0.0352). Although the numerical differences appear small, they are statistically significant, with TM_opt_ showing slightly higher activity than MW_opt_.

However, regarding the concentration required to achieve 50% inhibition (IC_50_), MW_opt_ showed lower values compared to TM_opt_, indicating greater antioxidant capacity. This difference was highly statistically significant (MW_opt_ = 0.016 μg/mL, TM_opt_ = 0.019 μg/mL, *p* < 0.0001). Similarly, when expressed in Trolox Equivalents, TM_opt_ showed statistically significantly higher values than MW_opt_ (MW_opt_ = 0.49 ± 0.00 mg TE/g, TM_opt_ = 0.51 ± 0.00 mg TE/g, *p* = 0.0001).

Although the absolute values appear close, statistically significant differences exist between the antioxidant activities of the two optimized extracts obtained by MW and TM, particularly regarding Trolox Equivalents and IC_50_ values.

### 3.4. Cytotoxic Effects of S. canadensis Extracts

**Figure 7:**
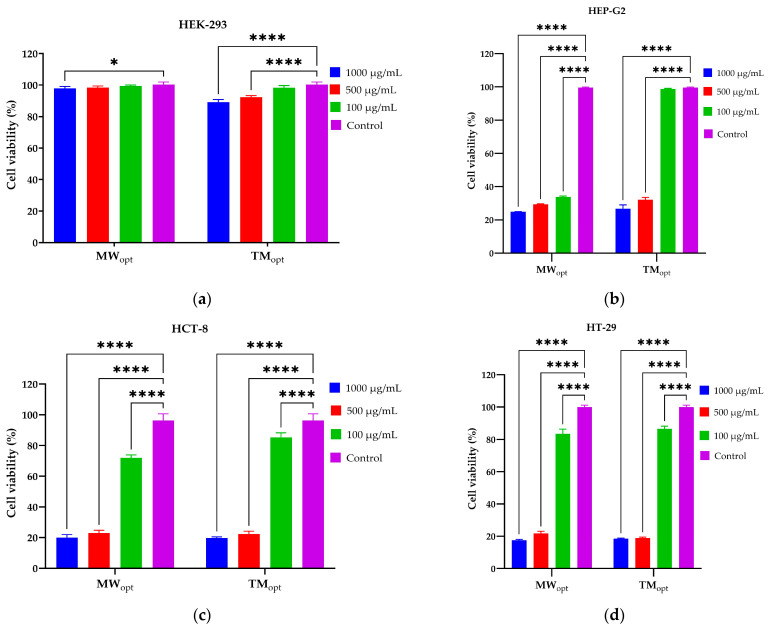
Cytotoxic effects in cells exposed for 24 h to various concentrations of *S. canadensis* extracts: (**a**) HEK-293, (**b**) HepG2, (**c**) HCT-8, and (**d**) HT-29. A two-way ANOVA followed by Tukey’s multiple comparisons test was used to compare the control with the experimental groups. Significant differences were noted at the following levels: * = *p* < 0.05, and **** = *p* < 0.0001.

The cytotoxic effects of *S. canadensis* extracts on HEK-293 (embryonic kidney) normal cells and HepG2 (liver), HCT-8 (colorectal), and HT-29 (colon) tumoral cells were examined using an MTT assay. In Figure 7, it was observed that treatment with different doses of *S. canadensis* extracts (100, 500, 1000 µg/mL) for 24 h resulted in a significant decrease in cell viability in HepG2 (Figure 7b), HCT-8 (Figure 7c), and HT-29 (Figure 7d) tumoral cells, particularly at doses of 500 and 1000 µg/mL. HepG2 cell viability decreased to 25% for the MW_opt_ extract and 26% for the TM_opt_ extract at a dose of 1000 µg/mL, compared to control cells. HCT-8 cell viability decreased to 20% for both MW_opt_ and TM_opt_ extracts at the same concentration, compared to control cells. HT-29 cell viability decreased to 17% for MW_opt_ extract and 18% for TM_opt_ extract at a dose of 1000 µg/mL, compared with control cells. Additionally, the results showed that *S. canadensis* extracts did not reduce cell viability in HEK-293 normal cells at the three doses tested (Figure 7a).

Table 9 shows IC_50_ values for *S. canadensis* extracts after 24 h of incubation. The MW_opt_ extract inhibited HepG2 cell growth by 50% (IC_50_ < 100 µg/mL) at doses of 100–1000 µg/mL, compared with control cells, whereas the TM_opt_ extract inhibited HepG2 cell growth by 50% (IC_50_ = 396.50 µg/mL) at higher doses of 500–1000 µg/mL, compared with control cells. HCT-8 and HT-29 tumor cells required similar doses of *S. canadensis* extracts (100–1000 µg/mL) to achieve comparable inhibition. For example, the MW_opt_ extract has IC_50_ values of 215.60 µg/mL in HCT-8 cells and 259.20 µg/mL in HT-29 cells. In addition, the TM_opt_ extract has IC_50_ values of 274.30 µg/mL in HCT-8 cells and 262.10 µg/mL in HT-29 cells.

**Figure 8:**
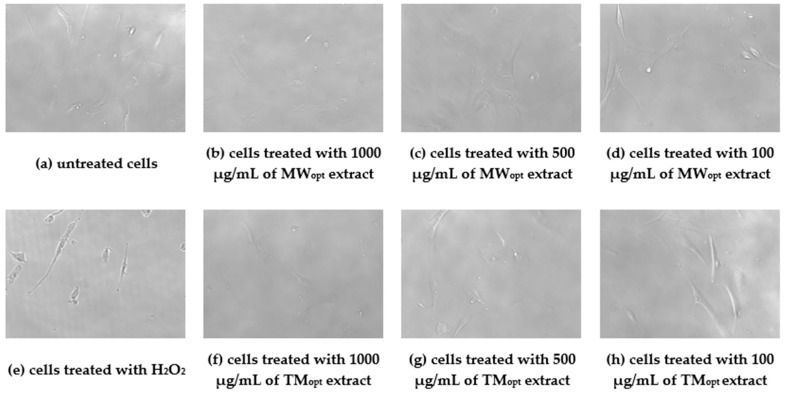
Cell morphology of (**a**) untreated HEK-293 cells (control negative) and (**e**) treated cells with H_2_O_2_ (control positive), (**b**–**d**) treated cells with MW_opt_ extract, and (**f**–**h**) treated cells with TM_opt_ extract.

**Figure 9:**
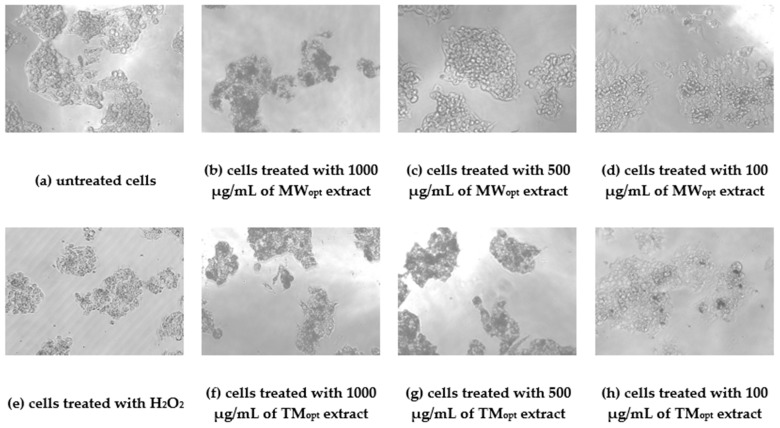
Cell morphology of (**a**) untreated HepG2 cells (control negative) and (**e**) treated cells with H_2_O_2_ (control positive), (**b**–**d**) treated cells with MW_opt_ extract, and (**f**–**h**) treated cells with TM_opt_ extract.

**Figure 10:**
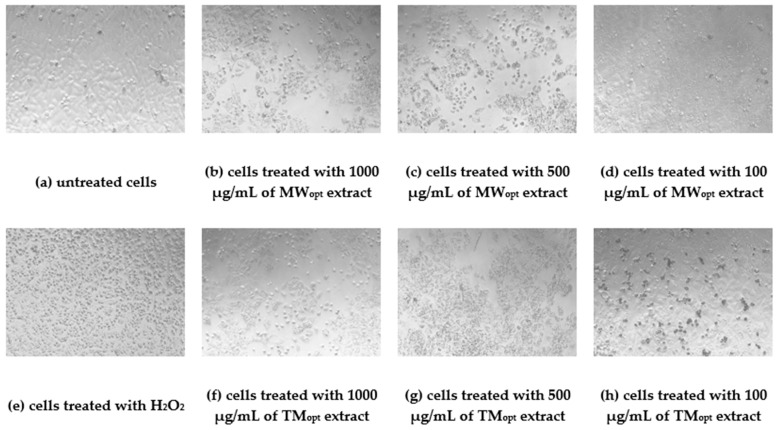
Cell morphology of (**a**) untreated HCT-8 cells (control negative) and (**e**) treated cells with H_2_O_2_ (control positive), (**b**–**d**) treated cells with MW_opt_ extract, and (**f**–**h**) treated cells with TM_opt_ extract.

**Figure 11:**
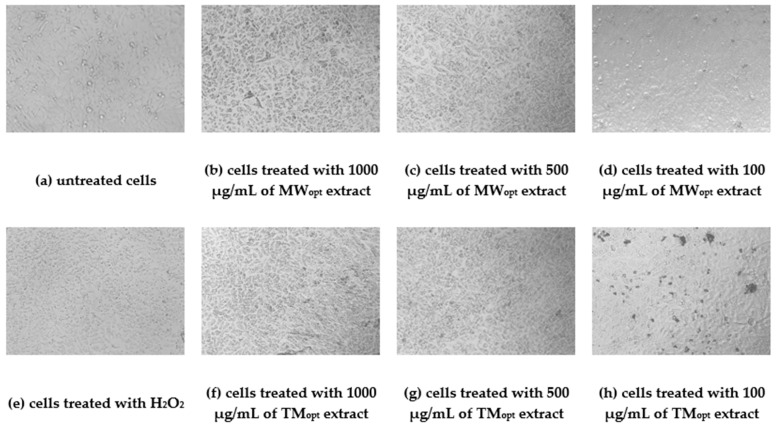
Cell morphology of (**a**) untreated HT-29 cells (control negative) and (**e**) treated cells with H_2_O_2_ (control positive), (**b**–**d**) treated cells with MW_opt_ extract, and (**f**–**h**) treated cells with TM_opt_ extract.

*S. canadensis* extracts do not induce morphological abnormalities in normal HEK-293 cells (Figure 8), as evidenced by the regular cell morphology observed in control cells, particularly in cells treated with the MWopt extract. Conversely, microscopic images of cell morphology indicated that *S. canadensis* extracts decreased cell viability and increased cell death in HepG2 (Figure 9), HCT-8 (Figure 10), and HT-29 (Figure 11) tumoral cells in a dose-dependent manner over a period of 24 h. 

### 3.5. Multivariate Statistical Analysis

**Figure 12:**
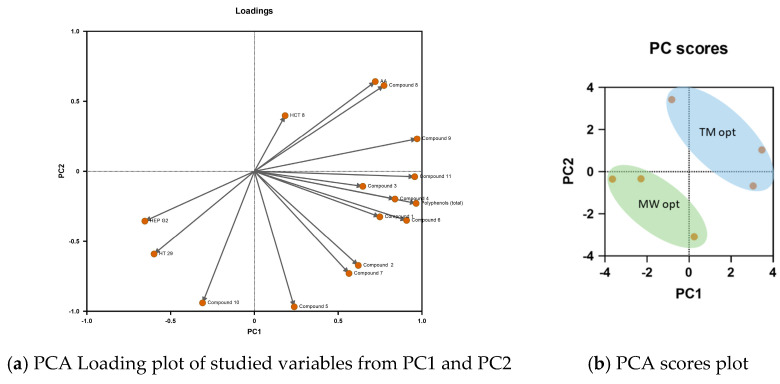
PCA of optimized extracts (Compound **1**: Dicaffeoylquinic acid 1 (mg CAE/g DW); Compound **2**: Neochlorogenic acid (mg/g DW); Compound **3**: Chlorogenic acid (mg/g DW); Compound **4**: Rutin (mg/g DW); Compound **5**: Quercetin-3-O-glucopyranoside (mg/g DW); Compound **6**: Kaempferol-3-O-rutinoside (mg/g DW); Compound **7**: Isorhamnetin-3-O-rutinoside (mg RE/g DW); Compound **8**: Dicaffeoylquinic acid 2 (mg CAE/g DW); Compound **9**: Flavonol (mg RE/g DW); Compound **10**: Dicaffeoylquinic acid 3 (mg CAE/g DW); Compound **11**: Flavonol (mg RE/g DW)).

Multivariate statistical analysis of the optimized *S. canadensis* extracts was performed using Principal Component Analysis (PCA). This method was used to highlight the similarities and differences between the results obtained for the two extracts, as well as to highlight the relationships between the analyzed variables. In this multivariate analysis, the following variables were considered: antioxidant activity, cytotoxic activity on the HepG2, HCT-8 and HT-29 cell lines, total sum of polyphenols, as well as the 11 phenolic compounds, measured by HPLC analysis. Information regarding the contribution of these variables to the first two principal components is presented in Figure 12, in the form of a loading plot of the studied variables.

The selection of the principal components was performed based on eigenvalues greater than 1, according to the Kaiser criterion. Thus, the first two principal components were retained for interpretation. The first principal component (PC1) explains 50.58% of the total variation in the data, while the second principal component (PC2) explains 28.79%, with the two components cumulatively accounting for 79.37% of the variance, indicating that they capture most of the variability in the analyzed dataset.

According to Figure 12a, PC1 is characterized by high positive loadings for the total sum of identified polyphenols and for eight specific phenolic compounds (compounds **1**–**4**, **6**, **7**, **9**, and **11**), suggesting that this component mainly reflects variations associated with the phenolic composition of the extracts. These variables contribute significantly to the separation of TMopt samples on the positive side of the PC1 axis. In contrast, cytotoxicity on HepG2 and HT-29 cell lines shows negative loadings on PC1, suggesting an inverse relationship between the concentration of phenolic compounds and the biological response of these cell lines. PC2 is mainly influenced by Compound **8**, antioxidant activity and cytotoxicity assessed on the HCT-8 cell line, variables that present high positive loadings and contribute to the separation of samples along this axis. Thus, PC2 highlights the specific differences between extracts in terms of certain phenolic components and associated biological activities.

Most of the phenolic compounds are positively correlated with each other, which suggests a similar chemical profile among the analyzed extracts. The distribution of variables in the loading plot indicates that PC1 is primarily associated with the chemical composition of the studied extracts, while PC2 reflects specific differences related to biological activity.

According to the PCA score plot (Figure 12b), the optimized extract MW_opt_ replicates showed a compact and homogeneous grouping, as indicated by the close proximity of the points. In fact, a clear difference was observed between the two extracts, TM_opt_ and MW_opt_. along with the PC1 and PC2 axes. Moreover, TMopt samples were associated with higher values of phenolic compounds and antioxidant activity, while MWopt samples are positioned opposite, reflecting differences in both the chemical composition and the evaluated biological activity. A reduced dispersion of the samples along PC2 indicates a good reproducibility of the analyses performed.

## 4. Discussion

Plants are a major source of biologically active compounds. Flavonoids and terpenes found in many medicinal species attract significant interest and possess chemopreventive and chemotherapeutic effects [48].

The comprehensive phytochemical characterization of Canadian goldenrod extracts by FTIR, HPLC, LC-MS/MS, and GC-MS analyses highlights a structurally diverse array of bioactive compounds with significant pharmaceutical potential. The combined presence of hydroxycinnamic acids, flavonoids, terpenoids, and lipid-derived compounds suggests a multifunctional phytocomplex capable of exerting synergistic biological effects, particularly in the context of oxidative stress and inflammation-related disorders. This chemical complexity suggests that the biological activity of *S. canadensis* extracts is not attributable to a single compound class, but rather to a synergistic interaction between phenolic and terpenoid constituents.

The use of the Box–Behnken design allowed efficient estimation of main effects and interactions between variables while reducing the total number of experiments required [49]. The quadratic models generated for TFC were highly significant for both extraction methods (*p* < 0.0001), confirming a good model fit. A strong correlation between experimental and predicted values was also observed.

Slightly superior statistical performance was obtained for TM, as indicated by higher R^2^ (0.9899), adjusted R^2^ (0.9769), predicted R^2^ (0.9125), and Adeq Precision (34.0860), together with a lower coefficient of variation (CV = 1.58%) compared to MW. Temperature and plant-to-solvent ratio significantly influenced TFC in both methods, while solvent concentration had a significant effect only in TM (*p* < 0.001), but not in MW (*p* > 0.05).

The extraction conditions were selected based on the operational characteristics of each method. Microwave-assisted extraction is generally characterized by shorter extraction times due to enhanced solvent penetration, rapid volumetric heating, and improved mass transfer. In contrast, traditional maceration relies on slower heat transfer and gradual solvent diffusion into plant tissues, resulting in reduced mass transfer efficiency and longer equilibrium times. Consequently, shorter extraction durations in TM (e.g., 1 h) may lead to incomplete recovery of bioactive compounds and lower overall yields.

The interaction between temperature and plant-to-solvent ratio was highly significant in both methods (*p* < 0.001), whereas the interaction between plant-to-solvent ratio and solvent concentration was not significant. These results indicate a higher predictive robustness of TM, while MW appears less dependent on solvent concentration.

Despite the slightly better predictive performance of TM, higher TFC values were obtained using MW under optimized conditions. This finding is consistent with previous studies, in which microwave-assisted extraction outperformed conventional maceration in *Cassia alata*, Sarawak Liberica coffee pulp, and *Stevia rebaudiana*, respectively [50,51,52].

Compared to liquid extracts, freeze-dried products exhibit higher physicochemical stability and increased concentrations of bioactive compounds, supporting their application in functional and therapeutic formulations. Lyophilization is widely regarded as a “gold standard” post-extraction technique due to its ability to remove water under low temperature and vacuum conditions, thereby minimizing thermal degradation and preserving thermolabile compounds [53].

Low processing temperatures reduce oxidative and enzymatic degradation, while ice crystal formation during freezing disrupts plant cell structures, enhancing the release and stabilization of intracellular phenolics. This contributes to improved retention of antioxidant activity compared to conventional drying methods [54]. Therefore, the combination of optimized extraction and lyophilization provides a dual advantage: maximized recovery and enhanced stability of bioactive constituents.

In the FTIR spectra of *S. canadensis* extracts, the broad band observed at 3600–3200 cm^−1^ corresponds to O–H stretching vibrations of hydroxyl-containing compounds, particularly phenols, alcohols, and carboxylic acids involved in hydrogen bonding [55]. The spectral region between 1115 and 1020 cm^−1^ supports the presence of C–O and C–O–C stretching vibrations associated with alcohols, ethers, esters, and carbohydrate groups [56]. These spectral features are characteristic of plant extracts and indicate the presence of phenolic groups and oxygenated moieties. In *S. canadensis*, the literature reports confirm that the aerial parts are rich in phenolic and flavonoid derivatives, compounds characterized by abundant O–H groups and C–O/C–O–C bonds [2]. Therefore, the FTIR spectra are in agreement with the results obtained by HPLC and LC-MS/MS analyses. This spectral consistency supports the reliability of the chemical profiling and confirms the predominance of oxygenated phenolic structures in the extracts.

Furthermore, the bands observed at 3000–2800 cm^−1^ and 1455–1400 cm^−1^ correspond to C–H stretching and bending vibrations of aliphatic CH_2_ and CH_3_ groups, indicating that, in addition to polyphenolic compounds, the extracts also contain hydrocarbon moieties or alkyl/methoxyl substituents [57]. This observation further supports the presence of volatile and semi-volatile constituents identified by GC-MS analysis. The low-intensity band in the 650–600 cm^−1^ region may be attributed to out-of-plane bending vibrations of phenolic groups or C–OH bonds, providing complementary structural evidence.

GC-MS analysis of MW_opt_ and TM_opt_ extracts revealed a wide range of volatile and semi-volatile compounds, including monoterpenes, oxygenated monoterpenes, sesquiterpenes, oxygenated sesquiterpenes, diterpenes, triterpenes, aromatic compounds and their derivatives, as well as fatty acids and esters. These findings highlight the rich phytochemical diversity and significant terpenoid content of *S. canadensis* extracts.

In essential oils from different *S. canadensis* species, D-limonene has been consistently reported as a major constituent. For example, Zhang et al. (2019) [58] identified D-limonene at abundances of 5.57% in *S. tortifolia* and 9.16% in *S. glomerata*. Similarly, Amtmann (2010) [59] reported the presence of DL-limonene (4.10%) and l-bornyl acetate (14.10%) in *S. canadensis* flowers. More recently, Fursenco et al. (2025) [5] reported D-limonene as a major compound (22.81%) in *S. canadensis*, along with a high content of α-pinene (28.80%) in *S. virgaurea* essential oils.

GC-MS analysis revealed a complementary profile of volatile and semi-volatile constituents, predominantly monoterpenes, sesquiterpenes, and their oxygenated derivatives, including D-limonene, carvone, verbenone, caryophyllene oxide, and spathulenol. These compounds are known for their antimicrobial, antioxidant, and anti-inflammatory activities [60,61]. D-limonene has been extensively studied for its anticancer properties, including apoptosis induction and tumor growth inhibition, while caryophyllene oxide exhibits anti-inflammatory effects [60]. The presence of diterpenes and triterpenes such as phytol and lupeol further strengthens the pharmacological relevance, as these compounds exhibit anti-inflammatory, hepatoprotective, and anticancer activities [62]. These results suggest that the volatile fraction contributes not only to antioxidant potential but may also enhance the cytotoxic profile through membrane interaction and apoptosis-related mechanisms.

Flavonoids, particularly quercetin and kaempferol derivatives, are the predominant components of the polyphenolic profile of *Solidago* spp. [63]. In the present study, hydroxycinnamic acids and flavonoids represent the main contributors to redox activity, suggesting that antioxidant effects are primarily driven by phenolic hydroxyl density and conjugation patterns.

HPLC and LC-MS/MS analyses consistently revealed hydroxycinnamic acids, including chlorogenic, neochlorogenic, dicaffeoylquinic, p-coumaric, and ferulic acids, as major constituents. These compounds are widely recognized for their potent antioxidant properties, acting as effective scavengers of reactive oxygen species (ROS) and modulators of redox-sensitive pathways [64]. Dicaffeoylquinic acids, in particular, have demonstrated strong antioxidant, antiviral, and hepatoprotective activities, while chlorogenic acid is associated with cardioprotective and antidiabetic effects [65]. Ferulic and p-coumaric acids further contribute through anti-inflammatory, antimicrobial, and chemopreventive mechanisms [66].

The flavonoid fraction, dominated by rutin, quercetin, isorhamnetin, luteolin, and naringenin derivatives, represents another key contributor to the pharmacological potential of the extracts. These compounds exhibit broad-spectrum bioactivities, including antioxidant, anti-inflammatory, antiviral, and anticancer effects [67]. Quercetin and its glycosides are known to modulate signaling pathways, leading to inhibition of tumor growth and inflammation [68]. Rutin contributes to vascular protection and capillary stability, while isorhamnetin displays improved bioavailability and cardioprotective effects [67]. Luteolin and naringenin further enhance the anti-inflammatory and neuroprotective potential of the extracts through cytokine modulation and enzyme inhibition [69].

To contextualize these findings within the broader genus, Toiu et al. (2019) [70] reported that extracts from the aerial parts of *S. graminifolia* contain flavonoid aglycones (quercetin, luteolin, and kaempferol), flavonoid glycosides (hyperoside, rutin, and quercitrin), and phenolic acids (caftaric, gentisic, chlorogenic, p-coumaric, ferulic, gallic, protocatechuic, vanillic, syringic, and rosmarinic acids). In the aerial parts of *S. virgaurea* var. *gigantea*, Jang et al. (2020) [71] identified and purified four polyphenolic compounds, namely 3,5-di-caffeoylquinic acid, protocatechuic acid, chlorogenic acid, and kaempferol-3-O-rutinoside. Further, a previous study reported by Ma et al. (2025) [72] indicates that the 10 most abundant polyphenolic compounds in *S. decurrens* are rutin, chlorogenic acid, D-(-)-quinic acid, kaempferol-3-O-rutinoside, quercetin, kaempferol, quercetin-3β-D-glucoside, L-(-)-malic acid, neochlorogenic acid, and isochlorogenic acid B.

Although TFC was higher in MW_opt_ than in TM_opt_, the TM_opt_ extract contained higher amounts of hydroxycinnamic acids, including dicaffeoylquinic acids and chlorogenic acid derivatives. These compounds are recognized as potent antioxidants due to their multiple hydroxyl groups and conjugated systems, and their higher concentration in TM_opt_ likely accounts for its superior antioxidant activity as measured by % DPPH inhibition and Trolox equivalents. Although MW_opt_ showed a slightly lower IC_50_ value, TM_opt_ exhibited statistically significantly higher values for both percentage inhibition and Trolox equivalents (*p* = 0.0352 and *p* = 0.0001, respectively). These findings suggest that flavonoids and hydroxycinnamic acids act synergistically in the antioxidant mechanism, and that the relative contribution of phenolic acid fractions can outweigh that of flavonoids when present in higher concentrations. The percentage of inhibition, IC_50_, and Trolox equivalents are complementary indicators of antioxidant potential as measured by the DPPH assay. Compared with the literature, optimized extraction of strawberry leaves by MW led to lower DPPH radical inhibitory percentages (79.80%) than the present study (85.36%) [73].

The HPLC and LC-MS/MS analyses showed that *S. canadensis* extracts contain a higher amount of hydroxycinnamic acids. Hydroxycinnamic acids show higher antioxidant activity than benzoic ones. Hydroxycinnamic acids, including caffeic acid, ferulic acid, p-coumaric acid, and chlorogenic acid, serve mainly as antioxidants by neutralizing reactive oxygen and nitrogen species. Their activity relies on phenolic hydroxyl and methoxy groups attached to the aromatic ring, which facilitate electron delocalization and stabilize radicals formed during the antioxidant process. The number and placement of –OH and –OCH_3_ groups indicate the compounds’ antioxidant effectiveness [65,74]. The primary mechanism is hydrogen atom transfer (HAT), where a hydroxycinnamic acid donates a hydrogen atom from its phenolic –OH to a free radical. Alternatively, they can undergo single-electron transfer followed by proton transfer (SET-PT), in which the phenolic compound first transfers an electron, then a proton [75,76,77]. After donating hydrogen or electrons, hydroxycinnamic acids produce phenoxyl radicals, stabilized by resonance as the unpaired electron delocalizes into the aromatic ring and conjugated side chain, reducing reactivity compared to the first radical. Specifically, ferulic acid’s phenoxyl radical can undergo coupling, dimerization, or neutralization with other radicals, thereby enhancing its antioxidant capacity [78].

The increased biological activity of the MW_opt_ and TM_opt_ extracts is mainly attributable to quercetin and its derivatives, and hydroxycinnamic acids. Quercetin and its derivatives have antioxidant, anti-inflammatory, and chemoprotective effects, linked to the prevention of oxidative stress and tumors. Their antioxidant activity stems from a polyphenolic structure rich in hydroxyl groups that donate hydrogen atoms or electrons to free radicals, thereby forming stabilized phenoxy radicals. Quercetin’s efficiency is enhanced by the catechol nucleus in the B ring, the conjugated C2=C3 double bond with the 4-oxo group, and free hydroxyl groups for electron delocalization and radical stabilization [79,80].

The cytotoxicity results demonstrated that *S. canadensis* extracts, MW_opt_, and TM_opt_ reduced the viability of HepG2 (liver), HCT-8 (colorectal), and HT-29 (colon) tumor cells in a dose-dependent manner over 24 h, without affecting HEK-293 (embryonic kidney) normal cells. The IC_50_ values and cell viability data showed that HCT-8 and HT-29 cells were more sensitive to the TM_opt_ extract than HepG2 cells. Moreover, both IC_50_ and cell viability values indicated that all tested tumor cell lines were sensitive to the MW_opt_ extract. Another study [39] reported the antiproliferative effects of *S. chilensis* extracts, as well as their phytochemical constituents, quercitrin and solidagenone, against five human tumor cell lines (U-251—glioblastoma, MCF-7—breast, 786-O—kidney, NCI-H460—non-small cell, and PC-3—prostate). These findings indicate a selective cytotoxic effect potentially associated with phenolic and terpenoid synergy, particularly involving flavonoid-mediated apoptosis and terpene-induced membrane disruption.

The MW_opt_ extract exhibited higher flavonoid content than the TM_opt_ extract, which may relate to its increased cytotoxicity effects [81]. MW extraction employs rapid, uniform heating, resulting in cell wall rupture and enhanced release of bioactive compounds [82]. In contrast, traditional extraction (TM) involves prolonged heat exposure, which can degrade heat-sensitive compounds [83]. Consequently, MW extraction yields greater amounts of phenolic and flavonoid compounds in less time [84]. The higher flavonoid concentration in the MW_opt_ extract is likely responsible for its cytotoxic effects through mechanisms such as induction of oxidative stress, modulation of apoptosis, inhibition of cell proliferation, and disruption of the cell cycle [85]. Nevertheless, cytotoxicity is also influenced by other bioactive compounds, as plant extracts contain diverse secondary metabolites that may act synergistically.

**Figure 13:**
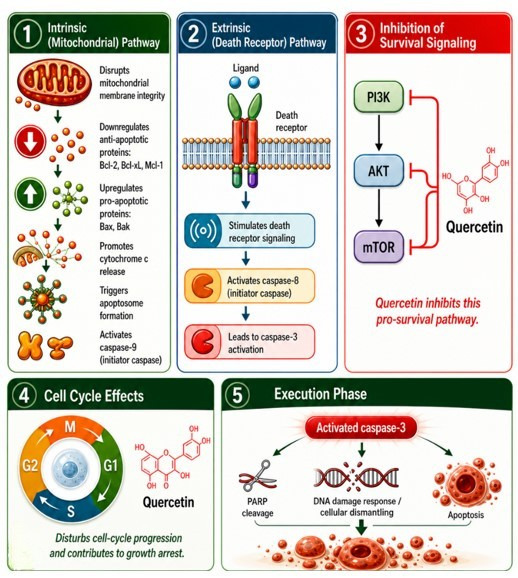
Possible mechanism of action for quercetin in the reduction in tumor cell viability.

Quercetin inhibits pathways such as PI3K/Akt/mTOR, MAPK/ERK, NF-κB, and JAK/STAT, thereby reducing proliferation and inducing apoptosis [86]. A possible mechanism of action for quercetin is presented in Figure 13. Rutin, a glycoside of quercetin found in *S. canadensis* extracts, contributes to antioxidant and antitumor effects. Through glycosylation, rutin also scavenges free radicals and reduces oxidative stress [87]. Regarding cytotoxic effects, rutin also regulates the same pathways as quercetin, promoting apoptosis and halting tumor growth [88].

Given the synergistic effects of flavonoids with phenolic compounds, the hydroxycinnamic acids identified in *S. canadensis* extracts are also associated with antioxidant, anti-inflammatory, and antitumoral activities because their hydroxyl groups allow hydrogen or electron donation and stabilize the resulting phenoxy radical [89,90]. Dicaffeoylquinic acids may play an important role because they contain two caffeoyl units, which may increase the capacity to neutralize radicals and support the cellular antioxidant response. The literature indicates that dicaffeoylquinic derivatives act not only by directly scavenging radicals but also by inducing the expression of antioxidant defense proteins, such as SOD, GPX, and CAT [91]. p-Coumaric acid and ferulic acid complete this profile, because they exhibit radical-scavenging activity, reducing capacity, and effects on Nrf2/HO-1 [92], while ferulic acid can inhibit tumor cell proliferation and induce apoptosis/autophagy in HepG2 cells [93].

These mechanisms suggest that the *S. canadensis* extracts may reduce tumor cell viability mainly through apoptosis-related pathways, particularly the intrinsic mitochondrial pathway.

According to the PCA, a clear separation was highlighted between the two extracts, MW_opt_ and TM_opt_, mainly due to the differences in the composition of phenolic compounds. Thus, the variability between replicates of the same extract was more visible on PC2, while the difference between the two extraction methods was mainly determined by PC1. TM_opt_ presented slightly higher concentrations for the main compounds on the positive side of PC1, whereas MW_opt_ was associated with higher values for compounds located on the negative side of PC1. A strong correlation between phenolic composition and antioxidant/cytotoxic responses was revealed by PCA, highlighting specific hydroxycinnamic acids and flavonoids as major contributors to bioactivity. Taken together, PCA further indicates that the extraction method significantly modulates the phytochemical profile, which subsequently governs the observed biological performance.

The coexistence of multiple compound classes supports their prospective application as natural antioxidant, anti-inflammatory, antimicrobial, and chemopreventive agents. These findings provide a strong rationale for further in vitro and in vivo studies aimed at elucidating mechanisms of action, bioavailability, and clinical relevance.

Although this study provides a comprehensive comparison of microwave-assisted and thermal maceration extraction methods combined with lyophilization, several limitations should be acknowledged. First, freeze-drying was applied exclusively as a preservation step to ensure sample stability prior to analysis; however, the potential influence of lyophilization on long-term phytochemical stability and bioactivity was not experimentally evaluated. Future investigations could address the stability of freeze-dried extracts under different storage conditions to further confirm their robustness. Second, the biological evaluation was limited to a selected panel of in vitro assays, including antioxidant activity and cytotoxicity on a restricted number of cancer cell lines. Furthermore, mechanistic studies were not performed to confirm the molecular pathways underlying the observed biological effects. As a result, the reported activities should be considered preliminary and require further validation through additional in vitro and in vivo studies. Finally, although plant material was collected under standardized conditions, the phytochemical composition of *S. canadensis* may vary according to environmental factors, geographical origin, harvesting period, and developmental stage, which may influence the reproducibility and generalizability of the results.

## 5. Conclusions

*Solidago canadensis* aerial parts were investigated, addressing the limited optimization of procedures aimed at maximizing polyphenolic recovery. Thermal maceration (TM) and microwave-assisted extraction (MW), followed by concentration and lyophilization, were applied to obtain stable, phenolic-enriched extracts suitable for chemical and biological evaluation.

A comprehensive phytochemical characterization (FTIR, HPLC, LC-MS/MS, and GC-MS) confirmed the presence of a chemically diverse phytocomplex composed of hydroxycinnamic acids, flavonoids, terpenoids, and lipid-derived constituents. This compositional complexity suggests a potential synergistic contribution to the observed biological effects.

Response surface methodology based on a Box–Behnken design provided statistically robust models for total flavonoid content. Although TM exhibited slightly superior model predictability, MW resulted in higher flavonoid yields under optimized conditions, indicating method-dependent differences in extraction performance.

Antioxidant and cytotoxic activities were closely associated with the phenolic profile, particularly hydroxycinnamic acids and flavonoids, as supported by multivariate (PCA) analysis. These findings indicate that extraction conditions significantly influence both phytochemical composition and bioactivity outcomes. This highlights the direct link between the extraction strategy and the biological performance of the resulting extracts.

MW demonstrated higher extraction efficiency, whereas TM showed greater model predictability, highlighting complementary advantages of the two approaches. Lyophilization further enhanced extract stability and applicability through the preservation of thermolabile compounds.

Therefore, the integration of optimized extraction, freeze-drying, and multilevel chemical–biological characterization enables the production of standardized *S. canadensis* extracts with promising antioxidant and cytotoxic potential, supporting further investigation for pharmaceutical, nutraceutical, and functional food applications.

## Figures and Tables

**Table 1:** Independent factors used in Box–Behnken Design.

Independent Factor	Factor Level		
	−1 (Low)	0	+1 (High)
Temperature (°C)	70	97.5	125
Plant to solvent ratio (w/v)	1:20	1:30	1:40
Solvent concentration (%)	50	60	70

**Table 2:** Experimental design in terms of independent factors.

Run	Temperature (°C)		Plant to Solvent Ratio (w/v)		Solvent Concentration (%)	
	Thermal Maceration	Microwave-Assisted Extraction	Thermal Maceration	Microwave-Assisted Extraction	Thermal Maceration	Microwave-Assisted Extraction
1	97.5	125	1:30	1:20	60	60
2	97.5	97.5	1:20	1:20	70	70
3	97.5	97.5	1:30	1:30	60	60
4	125	70	1:30	1:30	70	50
5	125	97.5	1:20	1:30	60	60
6	97.5	97.5	1:40	1:30	50	60
7	70	97.5	1:20	1:40	60	50
8	125	97.5	1:30	1:40	50	70
9	97.5	97.5	1:20	1:20	50	50
10	70	70	1:40	1:20	60	60
11	97.5	125	1:40	1:40	70	60
12	97.5	70	1:30	1:30	60	70
13	97.5	97.5	1:30	1:30	60	60
14	70	97.5	1:30	1:30	70	60
15	125	70	1:40	1:40	60	60
16	70	125	1:30	1:30	50	70
17	97.5	125	1:30	1:30	60	50

**Table 3:** Total flavonoid content of extracts.

Run	TFC (mg QE/g DW)	
	MW	TM
1	16.09 ± 0.30	15.14 ± 0.18
2	16.26 ± 0.41	13.45 ± 0.36
3	18.72 ± 0.56	15.43 ± 0.54
4	18.33 ± 0.64	15.85 ± 0.33
5	18.60 ± 1.62	12.05 ± 0.60
6	18.67 ± 0.74	15.87 ± 0.50
7	16.91 ± 0.33	13.96 ± 0.39
8	16.50 ± 0.63	13.55 ± 0.16
9	15.43 ± 0.18	12.69 ± 0.21
10	10.09 ± 0.61	15.21 ± 0.40
11	13.95 ± 0.35	16.95 ± 0.87
12	14.21 ± 0.16	15.14 ± 1.09
13	18.90 ± 0.77	15.63 ± 0.30
14	18.06 ± 0.04	14.28 ± 0.43
15	16.58 ± 0.38	18.44 ± 0.71
16	19.51 ± 0.52	14.55 ± 0.71
17	16.73 ± 0.32	15.51 ± 0.03

**Table 4:** ANOVA for quadratic models and model fit statistic.

Source	TFC (*p*-Value) MW	TFC (*p*-Value) TM
Model	**<0.0001**	**<0.0001**
A (Temperature)	**0.0014**	**0.0250**
B (Plant to solvent ratio)	**0.0032**	**<0.0001**
C (Solvent concentration)	0.5256	**0.0007**
AB	**<0.0001**	**<0.0001**
AC	**0.0002**	**0.0009**
BC	0.2445	0.5183
A^2^	**0.0002**	**0.0276**
B^2^	**<0.0001**	0.2760
C^2^	0.1846	**0.0036**
Lack of Fit	**0.0991**	**0.3939**
R^2^	0.9812	0.9899
Adjusted R^2^	0.9570	0.9769
Predicted R^2^	0.7640	0.9125
Adeq Precision	24.8717	34.0860
C.V. %	2.9300	1.5800

Values in bold indicate statistically significant differences (*p* < 0.05).

**Table 5:** Model validation.

	MW			TM		
TFC (mg QE/g DM)	Predicted	Experimental	*p* = 0.878	Predicted	Experimental	*p* = 0.912
	19.89	19.97 ± 0.52		18.87	18.78 ± 0.82	

**Table 6:** Phytochemical functional groups in the *S. canadensis* extracts by FTIR spectroscopy.

No.	Functional Groups	Stretching/Bending Vibrations	Range cm^−1^	MW_opt_	TM_opt_
1	Hydroxyl compounds	O–H stretch	3600–3200	+	+
2	Alkyl moieties	C–H stretch	3000–2800	+	+
3		C-H bend	1455–1400	+	+
4	Alcohols/Ethers	C–O/C-O-C stretch	1115–1020	+	+
5	Phenols	out-of-plane C-H bend	650–600	+	+

**Table 7:** Volatile and semi-volatile compounds in *S. canadensis* liofilized extracts.

No.	RT (min)	Compound Name	Area (%)	
			MW_opt_	TM_opt_
1	13.85	D-Limonene	0.72	-
2	15.01	(-)-cis-Verbenol	2.59	2.47
3	16.49	Catechol	2.46	6.37
4	16.95	Verbenone, (L)	1.52	1.67
5	17.21	Coumaran	2.05	2.04
6	17.30	cis-Carveol	1.56	1.60
7	18.06	Carvone	0.68	0.77
8	19.28	Bornyl acetate	2.32	1.59
9	20.00	4-Hydroxy-2-methylacetophenone	4.53	4.04
10	21.20	Eugenol	2.20	2.19
11	21.92	4-Ethylcatechol	3.24	2.82
12	24.62	4-(2,4,4-Trimethyl-cyclohexa-1,5-dienyl)-but-3-en-2-one	0.69	0.60
13	26.71	3-Hydroxymethylene-1,7,7-trimethylbicyclo[2.2.1]heptan-2-one	0.71	0.86
14	28.24	2-[4-Methyl-6-(2,6,6-trimethylcyclohex-1-enyl)hexa-1,3,5-trienyl]cyclohex-1-en-1-carboxaldehyde	0.80	0.78
15	28.52	Spathulenol	1.53	1.25
16	30.05	Andrographolide	1.79	1.49
17	31.81	Caryophyllene oxide	1.88	1.74
18	33.03	endo-1,5,6,7-Tetramethylbicyclo[3.2.0]hept-6-en-3-ol	-	0.65
19	33.22	2-Butenal, 2-methyl-4-(2,6,6-trimethyl-1-cyclohexen-1-yl)-	3.36	3.35
20	33.95	Platambin	1.65	1.55
21	34.15	2-Methyl-4-(2,6,6-trimethylcyclohex-1-enyl)but-2-en-1-ol	1.08	1.13
22	35.08	Hexadecanoic acid, methyl ester	11.04	8.10
23	35.38	2-[4-Methyl-6-(2,6,6-trimethylcyclohex-1-enyl)hexa-1,3,5-trienyl]cyclohex-1-en-1-carboxaldehyde	0.88	0.65
24	36.98	5β,7β-H,10α-Eudesm-11-en-1α-ol	0.75	-
25	38.45	Linoleic acid, methyl ester	4.42	2.89
26	38.56	Linolenic acid, methyl ester	5.20	3.28
27	38.76	Phytol	2.22	2.01
28	39.09	Methyl stearate	5.06	3.85
29	42.73	6-epi-Shyobunol	0.82	0.90
30	43.20	Lupeol	0.86	1.07

RT (min) = Retention Time (minutes).

**Table 8:** Polyphenolic compounds identified in *S. canadensis* extracts by LC-MS/MS.

No.	Compound Name	Compound Class	[M−H]^−^ *m*/*z*	MS/MS Fragments	References
1	p-Coumaric acid	Hydroxycinnamic acid	163.0546	119.0495, 93.0538	[32,33]
2	Gallic acid	Hydroxybenzoic acid	169.1043	125.0245, 79.0296	[34,35,36]
3	Quinic acid	Cyclohexanecarboxylic acid	191.0543	111.0079, 87.0102	[35,37]
4	Ferulic acid	Hydroxycinnamic acid	193.1134	178.4229, 149.3121, 133.9999	[32,34]
5	Naringenin	Flavanone	271.0621	151.0050; 119.0510	[38,39]
6	Luteolin	Flavone	285.0394	151.0441, 133.0298	[40,41]
7	Quercetin	Flavonol	301.0347	178.9974, 151.0024, 121.0827	[33,34]
8	Isorhamnetin	O-methylated flavonol	315.0508	300.0248, 271.0244, 151.0036	[38,41]
9	p-Coumaroylquinic acid	Hydroxycinnamic acid derivatives	337.0916	191.0550, 163.0839	[42,43]
10	Chlorogenic acid	Hydroxycinnamic acid	353.0802	191.0496, 85.0249	[33,41]
11	Rutin	Flavonol glycoside	609.1303	301.0546	[41,44]

**Table 9:** IC_50_ values of *S. canadensis* extracts obtained after 24 h of incubation in the tested tumoral cell lines. Values were obtained from dose–response curves using GraphPad Prism Software version 10.5.0.

*S. canadensis* Extracts	HepG2	HCT-8	HT-29
MW_opt_	<100	215.60	259.20
TM_opt_	396.50	274.30	262.10

The results are expressed in µg/mL.

## Data Availability

The original contributions presented in this study are included in the article. Further inquiries can be directed to the corresponding authors.

## References

[1] Poljuha D., Sladonja B., Uzelac Božac M., Šola I., Damijanić D., Weber T. (2024). The invasive alien plant *Solidago canadensis*: Phytochemical composition, ecosystem service potential, and application in bioeconomy. Plants.

[2] Uzelac Božac M., Poljuha D., Dudaš S., Bilić J., Šola I., Mikulič-Petkovšek M., Sladonja B. (2025). Phenolic profile and antioxidant capacity of invasive *Solidago canadensis* L.: Potential applications in phytopharmacy. Plants.

[3] Shelepova O., Vinogradova Y., Vergun O., Grygorieva O., Brindza J. (2020). Assessment of flavonoids and phenolic compound accumulation in invasive *Solidago canadensis* L. in Slovakia. Potravin. Slovak J. Food Sci..

[4] Georgescu M.I., Cîşlariu A.G., Gîdea M., Săvulescu E. (2024). Preliminary data about the invasive ability of *Solidago canadensis* L. and its establishment in crops. Sci. Pap. Ser. A Agron..

[5] Fursenco C., Ion V.A., Calalb T., Uncu L. (2025). Comparative assessment of active compounds in Solidago species from the flora of the Republic of Moldova. Mold. J. Health Sci..

[6] European Directorate for the Quality of Medicines, HealthCare (EDQM) (2025). European Pharmacopoeia.

[7] Likhanov A., Oliinyk M., Pashkevych N., Churilov A., Kozyr M. (2021). The role of flavonoids in invasion strategy of *Solidago canadensis* L. Plants.

[8] Apáti P., Szentmihályi K., Kristó S.T., Papp I., Vinkler P., Szőke É., Kéry Á. (2003). Herbal remedies of Solidago—Correlation of phytochemical characteristics and antioxidative properties. J. Pharm. Biomed. Anal..

[9] Koshovyi O., Hrytsyk Y., Perekhoda L., Suleiman M., Jakštas V., Žvikas V., Grytsyk L., Yurchyshyn O., Heinämäki J., Raal A. (2025). *Solidago canadensis* L. herb extract, its amino acid preparations and 3D-printed dosage forms: Phytochemical, technological, molecular docking and pharmacological research. Pharmaceutics.

[10] Hrytsyk Y., Koshovyi O., Lepiku M., Jakštas V., Žvikas V., Matus T., Melnyk M., Grytsyk L., Raal A. (2024). Phytochemical and pharmacological research in galenic remedies of *Solidago canadensis* L. herb. Phyton.

[11] Jovanović A.A., Đorđević V.B., Zdunić G.M., Pljevljakušić D.S., Šavikin K.P., Gođevac D.M., Bugarski B.M. (2017). Optimization of the extraction process of polyphenols from *Thymus serpyllum* L. herb using maceration, heat- and ultrasound-assisted techniques. Sep. Purif. Technol..

[12] Altemimi A., Lakhssassi N., Baharlouei A., Watson D.G., Lightfoot D.A. (2017). Phytochemicals: Extraction, isolation, and identification of bioactive compounds from plant extracts. Plants.

[13] Bhadange Y.A., Carpenter J., Saharan V.K. (2024). A comprehensive review on advanced extraction techniques for retrieving bioactive components from natural sources. ACS Omega.

[14] Luksta I., Spalvins K. (2023). Methods for extraction of bioactive compounds from products: A review. Environ. Clim. Technol..

[15] Luthria D.L. (2008). Influence of experimental conditions on the extraction of phenolic compounds from parsley (*Petroselinum crispum*) flakes using a pressurized liquid extractor. Food Chem..

[16] Sun S., Yu Y., Jo Y., Han J.H., Xue Y., Cho M., Bae S.-J., Ryu D., Park W., Ha K.-T. (2025). Impact of extraction techniques on phytochemical composition and bioactivity of natural product mixtures. Front. Pharmacol..

[17] Shao P., He J., Sun P., Zhao P. (2011). Analysis of conditions for microwave-assisted extraction of total water-soluble flavonoids from *Perilla frutescens* leaves. J. Food Sci. Technol..

[18] Karami Z., Emam-Djomeh Z., Mirzaee H.A., Khomeiri M., Mahoonak A.S., Aydani E. (2015). Optimization of microwave-assisted extraction and Soxhlet extraction of phenolic compounds from licorice root. J. Food Sci. Technol..

[19] Dahmoune F., Nayak B., Moussi K., Remini H., Madani K. (2015). Optimization of microwave-assisted extraction of polyphenols from *Myrtus communis* L. leaves. Food Chem..

[20] Pan X., Niu G., Liu H. (2003). Microwave-assisted extraction of tea polyphenols and tea caffeine from green tea leaves. Chem. Eng. Process. Process Intensif..

[21] Mikucka W., Zielinska M., Bulkowska K., Witonska I. (2022). Recovery of polyphenols from distillery stillage by microwave-assisted, ultrasound-assisted and conventional solid–liquid extraction. Sci. Rep..

[22] Lozano Pérez A.S., Lozada Castro J.J., Guerrero Fajardo C.A. (2024). Application of Microwave Energy to Biomass: A Comprehensive Review of Microwave-Assisted Technologies, Optimization Parameters, and the Strengths and Weaknesses. J. Manuf. Mater. Process..

[23] López-Salazar H., Camacho-Díaz B.H., Ocampo M.L.A., Jiménez-Aparicio A.R. (2023). Microwave-assisted extraction of functional compounds from plants: A Review. BioResources.

[24] Marcu Spinu S., Dragoi Cudalbeanu M., Major N., Goreta Ban S., Palčić I., Ortan A., Rosu P.M., Babeanu N.E. (2025). Box–Behnken Design Optimization of Green Extraction from Tomato Aerial Parts and Axillary Shoots for Enhanced Recovery of Rutin and Complementary Bioactive Compounds. Antioxidants.

[25] Savic S., Petrovic S., Knezevic-Jugovic Z. (2026). Separation Strategies for Polyphenols from Plant Extracts: Advances, Challenges, and Applications. Separations.

[26] Coşkun N., Sarıtaş S., Jaouhari Y., Bordiga M., Karav S. (2024). The Impact of Freeze Drying on Bioactivity and Physical Properties of Food Products. Appl. Sci..

[27] Wang Y., Fu J., Yang D. (2021). In Situ Stability of Anthocyanins in *Lycium ruthenicum* Murray. Molecules.

[28] Al Hasani S., Al-Attabi Z., Waly M., Al-Habsi N., Al-Subhi L., Shafiur Rahman M. (2023). Polyphenol and Flavonoid Stability of Wild Blueberry (*Sideroxylon mascatense*) during Air- and Freeze-Drying and Storage Stability as a Function of Temperature. Foods.

[29] Chandra S., Khan S., Avula B., Lata H., Yang M.H., ElSohly M.A., Khan I.A. (2014). Assessment of total phenolic and flavonoid content, antioxidant properties, and yield of aeroponically and conventionally grown leafy vegetables and fruit crops: A comparative study. Evid. Based Complement. Altern. Med..

[30] Shraim A.M., Ahmed T.A., Rahman M.M., Hijji Y.M. (2021). Determination of total flavonoid content by aluminum chloride assay: A critical evaluation. LWT.

[31] Marcu Spinu S., Dragoi Cudalbeanu M., Avram I., Fierascu R.C., Rosu P.M., Morosanu A.-M., Cimpeanu C.L., Babeanu N., Ortan A. (2024). Antibacterial and Antitumoral Potentials of Phytosynthesized Silver/Silver Oxide Nanoparticles Using Tomato Flower Waste. Int. J. Mol. Sci..

[32] Bursal E., Köksal E., Gülçin İ., Bilsel G., Gören A.C. (2013). Antioxidant activity and polyphenol content of cherry stem (*Cerasus avium* L.) determined by LC–MS/MS. Food Res. Int..

[33] Pereira-Coelho M., da Silva Haas I.C., Reinke C.K., Dognini J., Amboni R.D.D.M.C., Vitali L., dos Santos Madureira L.A. (2023). A green analytical method for the determination of polyphenols in wine by dispersive pipette extraction and LC-MS/MS. Food Chem..

[34] Kumar Y., Singhal S., Tarafdar A., Pharande A., Ganesan M., Badgujar P.C. (2020). Ultrasound assisted extraction of selected edible macroalgae: Effect on antioxidant activity and quantitative assessment of polyphenols by liquid chromatography with tandem mass spectrometry (LC-MS/MS). Algal Res..

[35] Zheng H., Du H., Ye E., Xu X., Wang X., Jiang X., Min Z., Zhuang L., Li S., Guo L. (2024). Optimized extraction of polyphenols with antioxidant and anti-biofilm activities and LC-MS/MS-based characterization of phlorotannins from *Sargassum muticum*. LWT.

[36] Zouaoui Z., Ennoury A., El Asri S., Laabar A., Kabach I., Vinci R.L., Cacciola F., Mondello L., Taghzouti K., Nhiri M. (2025). Polyphenols from rose pepper spice: LC-MS/MS characterization and therapeutic potential in diabetes mellitus management. Food Biosci..

[37] Raskar S., Hooda R., Mukherjee A., Mitra S. (2025). LC-MS/MS-based metabolic profiling and in silico molecular modeling revealed a quorum sensing inhibitor from *Embelia ribes* Burm. f. fruits. Food Biosci..

[38] Chen Y., Yu H., Wu H., Pan Y., Wang K., Jin Y., Zhang C. (2015). Characterization and Quantification by LC-MS/MS of the Chemical Components of the Heating Products of the Flavonoids Extract in Pollen Typhae for Transformation Rule Exploration. Molecules.

[39] Orrego-Lagarón N., Vallverdu-Queralt A., Martínez-Huélamo M., Lamuela-Raventos R.M., Escribano-Ferrer E. (2016). Metabolic profile of naringenin in the stomach and colon using liquid chromatography/electrospray ionization linear ion trap quadrupole-Orbitrap-mass spectrometry (LC-ESI-LTQ-Orbitrap-MS) and LC-ESI-MS/MS. J. Pharm. Biomed. Anal..

[40] Desta K.T., Kim G.S., Abd El-Aty A.M., Raha S., Kim M.B., Jeong J.H., Warda M., Hacımüftüoğlu A., Shin H.-C., Shim J.-H. (2017). Flavone polyphenols dominate in *Thymus schimperi* Ronniger: LC–ESI–MS/MS characterization and study of anti-proliferative effects of plant extract on AGS and HepG2 cancer cells. J. Chromatogr. B.

[41] Gülçin İ., Gören A.C., Taslimi P., Alwasel S.H., Kılıc O., Bursal E. (2020). Anticholinergic, antidiabetic and antioxidant activities of Anatolian pennyroyal (*Mentha pulegium*)—Analysis of its polyphenol contents by LC-MS/MS. Biocatal. Agric. Biotechnol..

[42] Wen M., Han Z., Cui Y., Ho C.T., Wan X., Zhang L. (2022). Identification of 4-Op-coumaroylquinic acid as astringent compound of Keemun black tea by efficient integrated approaches of mass spectrometry, turbidity analysis and sensory evaluation. Food Chem..

[43] Lopez-Rodulfo I.M., Tsochatzis E.D., Stentoft E.W., Martinez-Carrasco P., Bechtner J.D., Martinez M.M. (2024). Partitioning and in vitro bioaccessibility of apple polyphenols during mechanical and physiological extraction: A hierarchical clustering analysis with LC-ESI-QTOF-MS/MS. Food Chem..

[44] El-Ghazouani F., Amri O., Bouhaimi A., Zekhnini A. (2025). Myricitrin, kaempferol-3-O-rutinoside, and rutin from *Acacia tortilis* (Forssk.) Hayne ssp. *raddiana* alleviate liver injury in carbon tetrachloride (CCl_4_)-intoxicated rats. S. Afr. J. Bot..

[45] Fabre N., Rustan I., de Hoffmann E., Quetin-Leclercq J. (2001). Determination of flavone, flavonol, and flavanone aglycones by negative ion liquid chromatography electrospray ion trap mass spectrometry. J. Am. Soc. Mass Spectrom..

[46] Grati W., Samet S., Bouzayani B., Ayachi A., Treilhou M., Téné N., Mezghani-Jarraya R. (2022). HESI-MS/MS analysis of phenolic compounds from *Calendula aegyptiaca* fruits extracts and evaluation of their antioxidant activities. Molecules.

[47] Jiang C., Gates P.J. (2024). Systematic characterisation of the fragmentation of flavonoids using high-resolution accurate mass electrospray tandem mass spectrometry. Molecules.

[48] Gomes D.B., Zanchet B., Locateli G., Benvenutti R.C., Vechia C.A.D., Schönell A.P., Diel K.A., Zilli G.A., Miorando D., Ernetti J. (2018). Antiproliferative potential of solidagenone isolated of *Solidago chilensis*. Rev. Bras. Farmacogn..

[49] Mostafa E.A., El-Ashrey M.K., Mahmoud S.T. (2023). An innovative combination of Box-Behnken design and ecofriendly approaches for the simultaneous determination of aspirin, clopidogrel, atorvastatin and rosuvastatin in their fixed-dose combination tablets. BMC Chem..

[50] Ling Y.Y., Fun P.S., Yeop A., Yusoff M.M., Gimbun J. (2019). Assessment of Maceration, Ultrasonic and Microwave Assisted Extraction for Total Phenolic Content, Total Flavonoid Content and Kaempferol Yield from *Cassia alata* via Microstructures Analysis. Mater. Today Proc..

[51] Wong J.C.J., Nillian E. (2023). Microwave-assisted extraction of bioactive compounds from Sarawak *Liberica* sp. coffee pulp: Statistical optimization and comparison with conventional methods. Food Sci. Nutr..

[52] Kumar P., Tripathi P.P. (2025). Comparative Evaluation and Optimization of Microwave and Ultrasound Assisted Extraction of Stevia Secondary Bioactive Compounds Using RSM and ANN–GA Approaches. Sustain. Food Technol..

[53] ElNaker N.A., Daou M., Ochsenkühn M.A., Amin S.A., Yousef A.F., Yousef L.F. (2021). A metabolomics approach to evaluate the effect of lyophilization versus oven drying on the chemical composition of plant extracts. Sci. Rep..

[54] Babaei Rad S., Mumivand H., Mollaei S., Khadivi A. (2025). Effect of drying methods on phenolic compounds and antioxidant activity of *Capparis spinosa* L. fruits. BMC Plant Biol..

[55] Bodea I.M., Cătunescu G.M., Pop C.R., Fiț N.I., David A.P., Dudescu M.C., Stănilă A., Rotar A.M., Beteg F.I. (2022). Antimicrobial Properties of Bacterial Cellulose Films Enriched with Bioactive Herbal Extracts Obtained by Microwave-Assisted Extraction. Polymers.

[56] Agatonovic-Kustrin S., Balyklova K.S., Gegechkori V., Morton D.W. (2021). HPTLC and ATR/FTIR Characterization of Antioxidants in Different Rosemary Extracts. Molecules.

[57] Kato-Noguchi H., Kato M. (2022). Allelopathy and Allelochemicals of *Solidago canadensis* L. and *S. altissima* L. for Their Naturalization. Plants.

[58] Zhang Y., Jia C., Zhang Y., Yang S., Dong Y., Wei D., Sun J., Wang S., He S., Li J. (2019). Chemical variability in volatile composition among five species of genus Solidago (Asteraceae). Biochem. Syst. Ecol..

[59] Amtmann M. (2010). The chemical relationship between the scent features of goldenrod (*Solidago canadensis* L.) flower and its unifloral honey. J. Food Compos. Anal..

[60] Miguel M.G. (2010). Antioxidant and anti-inflammatory activities of essential oils: A short review. Molecules.

[61] Legault J., Pichette A. (2007). Potentiating effect of β-caryophyllene on anticancer activity of α-humulene, isocaryophyllene and paclitaxel. J. Pharm. Pharmacol..

[62] Marchese A., Barbieri R., Coppo E., Orhan I.E., Daglia M., Nabavi S.F., Izadi M., Abdollahi M., Nabavi S.M., Ajami M. (2017). Antimicrobial activity of eugenol and essential oils containing eugenol: A mechanistic viewpoint. Crit. Rev. Microbiol..

[63] Marksa M., Zymone K., Ivanauskas L., Radušienė J., Pukalskas A., Raudone L. (2020). Antioxidant profiles of leaves and inflorescences of native, invasive and hybrid Solidago species. Ind. Crops Prod..

[64] Shahidi F., Ambigaipalan P. (2015). Phenolics and polyphenolics in foods, beverages and spices: Antioxidant activity and health effects—A review. J. Funct. Foods.

[65] Kumar N., Goel N. (2019). Phenolic acids: Natural versatile molecules with promising therapeutic applications. Biotechnol. Rep..

[66] Heleno S.A., Martins A., Queiroz M.J.R.P., Ferreira I.C.F.R. (2015). Bioactivity of phenolic acids: Metabolites versus parent compounds: A review. Food Chem..

[67] Pietta P.-G. (2000). Flavonoids as antioxidants. J. Nat. Prod..

[68] Dabeek W.M., Marra M.V. (2019). Dietary quercetin and kaempferol: Bioavailability and potential cardiovascular-related bioactivity in humans. Nutrients.

[69] Aziz N., Kim M.-Y., Cho J.Y. (2018). Anti-inflammatory effects of luteolin: A review of in vitro, in vivo, and in silico studies. J. Ethnopharmacol..

[70] Toiu A., Vlase L., Vodnar D.C., Gheldiu A.-M., Oniga I. (2019). *Solidago graminifolia* L. Salisb. (Asteraceae) as a Valuable Source of Bioactive Polyphenols: HPLC Profile, In Vitro Antioxidant and Antimicrobial Potential. Molecules.

[71] Jang Y.S., Kim H., Zuo G., Lee E.H., Kang S.K., Lim S.S. (2020). Constituents from *Solidago virgaurea* var. *gigantea* and their inhibitory effect on lipid accumulation. Fitoterapia.

[72] Ma D., Peng L., Gao X., Xing T., Hao Z. (2025). *Solidago decurrens* Lour. Controls LPS-induced acute lung injury by reducing inflammatory responses and modulating the TLR4/NF-κB/NLRP3 signaling pathway. J. Ethnopharmacol..

[73] Lin D., Ma Q., Zhang Y., Peng Z. (2020). Phenolic compounds with antioxidant activity from strawberry leaves: A study on microwave-assisted extraction optimization. Prep. Biochem. Biotechnol..

[74] Moazzen A., Öztinen N., Ak-Sakalli E., Koşar M. (2022). Structure-antiradical activity relationships of 25 natural antioxidant phenolic compounds from different classes. Heliyon.

[75] Mazzone G., Russo N., Toscano M. (2016). Antioxidant properties comparative study of natural hydroxycinnamic acids and structurally modified derivatives: Computational insights. Comput. Theor. Chem..

[76] Tošović J., Marković S., Dimitrić Marković J.M., Mojović M., Milenković D. (2017). Antioxidative mechanisms in chlorogenic acid. Food Chem..

[77] Biela M., Kleinová A., Klein E. (2022). Phenolic acids and their carboxylate anions: Thermodynamics of primary antioxidant action. Phytochemistry.

[78] Amić A., Marković Z., Dimitrić Marković J.M., Milenković D., Stepanić V. (2020). Antioxidative potential of ferulic acid phenoxyl radical. Phytochemistry.

[79] Kumar S., Pandey A.K. (2013). Chemistry and Biological Activities of Flavonoids: An Overview. Sci. World J..

[80] Vollmannová A., Bojňanská T., Musilová J., Lidiková J., Cifrová M. (2024). Quercetin as one of the most abundant represented biological valuable plant components with remarkable chemoprotective effects—A review. Heliyon.

[81] Mir S.A., Dar A., Hamid L., Nisar N., Malik J.A., Ali T., Bader G.N. (2023). Flavonoids as promising molecules in the cancer therapy: An insight. Curr. Res. Pharmacol. Drug Discov..

[82] Carniel N., Dallago R.M., Dariva C., Bender J.P., Nunes A.L., Zanella O., Bilibio D., Luiz Priamo W. (2017). Microwave-Assisted Extraction of Phenolic Acids and Flavonoids from *Physalis angulata*. J. Food Process Eng..

[83] Hu L., Wang C., Guo X., Chen D., Zhou W., Chen X., Zhang Q. (2021). Flavonoid Levels and Antioxidant Capacity of Mulberry Leaves: Effects of Growth Period and Drying Methods. Front. Plant Sci..

[84] Zhang H., Zhang X., Yang X., Qiu N., Wang Y., Wang Z. (2013). Microwave assisted extraction of flavonoids from cultivated *Epimedium sagittatum*: Extraction yield and mechanism, antioxidant activity and chemical composition. Ind. Crops Prod..

[85] Ravishankar D., Rajora A.K., Greco F., Osborn H.M. (2013). Flavonoids as prospective compounds for anti-cancer therapy. Int. J. Biochem. Cell Biol..

[86] Silva-Pinto P.A., De Pontes J.T.C., Aguilar-Morón B., Canales C.S.C., Pavan F.R., Roque-Borda C.A. (2025). Phytochemical insights into flavonoids in cancer: Mechanisms, therapeutic potential, and the case of quercetin. Heliyon.

[87] Chekuri S., Sirigiripeta S.R., Thupakula S., Vyshnava S.S., Ayesha S., Karamthote Cheniya S.B., Kuruva R., Anupalli R.R. (2025). Rutin isolated from *Acalypha indica* L.: A comprehensive analysis of its antibacterial and anticancer activities. Biochem. Biophys. Res. Commun..

[88] Pandey P., Lakhanpal S., Mahmood D., Kang H.N., Kim B., Kang S., Choi J., Choi M., Pandey S., Bhat M. (2025). An updated review summarizing the anticancer potential of flavonoids via targeting NF-kB pathway. Front. Pharmacol..

[89] Wang L., Pan X., Jiang L., Chu Y., Gao S., Jiang X., Zhang Y., Chen Y., Luo S., Peng C. (2022). The Biological Activity Mechanism of Chlorogenic Acid and Its Applications in Food Industry: A Review. Front. Nutr..

[90] Huang J., Xie M., He L., Song X., Cao T. (2023). Chlorogenic acid: A review on its mechanisms of anti-inflammation, disease treatment, and related delivery systems. Front. Pharmacol..

[91] Hufnagel M., Rademaekers A., Weisert A., Häberlein H., Franken S. (2024). Pharmacological profile of dicaffeoylquinic acids and their role in the treatment of respiratory diseases. Front. Pharmacol..

[92] Shen Y., Song X., Li L., Sun J., Jaiswal Y., Huang J., Liu C., Yang W., Williams L., Zhang H. (2019). Protective effects of p-coumaric acid against oxidant and hyperlipidemia-an in vitro and in vivo evaluation. Biomed. Pharmacother..

[93] Wang J., Lai X., Yuan D., Liu Y., Wang J., Liang Y. (2022). Effects of ferulic acid, a major component of rice bran, on proliferation, apoptosis, and autophagy of HepG2 cells. Food Res. Int..

